# Multidimensional, integrative profiling identifies BCL2L1 methylation as a predictor of MCL1 dependency in pediatric malignancies

**DOI:** 10.1172/jci.insight.184601

**Published:** 2025-01-23

**Authors:** Shazia Adjumain, Paul Daniel, Claire Xin Sun, Gabrielle Bradshaw, Nicole J. Chew, Vanessa Tsui, Hanbyeol Lee, Melissa Loi, Nataliya Zhukova, Dilru Habarakada, Abigail Yoel, Vijesh G. Vaghjiani, Shaye Game, Louise E. Ludlow, Naama Neeman, E. Alejandro Sweet-Cordero, David D. Eisenstat, Jason E. Cain, Ron Firestein

**Affiliations:** 1 Centre for Cancer Research, Hudson Institute of Medical Research, and; 2 Department of Molecular and Translational Science, Faculty of Medicine, Nursing and Health Sciences, Monash University, Clayton, Victoria, Australia.; 3 Children’s Cancer Centre, Monash Children’s Hospital, Monash Health, Clayton, Victoria, Australia.; 4 Department of Pediatrics, Monash University, Clayton, Victoria, Australia.; 5 Children’s Cancer Centre Biobank, Murdoch Children’s Research Institute, Parkville, Victoria, Australia.; 6 Department of Pediatrics, School of Medicine, University of California, San Francisco, San Francisco, California, USA.; 7 Children’s Cancer Centre, The Royal Children’s Hospital Melbourne, Parkville, Victoria, Australia.; 8 Neuro-Oncology Group, Stem Cell Medicine, Murdoch Children’s Research Institute, Parkville, Victoria, Australia.; 9 Department of Pediatrics, University of Melbourne, Parkville, Victoria, Australia.

**Keywords:** Clinical trials, Therapeutics, Drug therapy

## Abstract

Pediatric high-grade gliomas (pHGGs) are the most aggressive brain tumors in children, necessitating innovative therapies to improve outcomes. Unlike adult gliomas, recent research reveals that childhood gliomas have distinct biological features, requiring specific treatment strategies. Here, we focused on deciphering unique genetic dependencies specific to childhood gliomas. Using a pooled CRISPR/Cas9 knockout screening approach on 65 pediatric and 10 adult high-grade glioma (HGG) cell lines, myeloid cell leukemia 1 (*MCL1*) emerged as a key antiapoptotic gene essential in pediatric but not adult gliomas. We demonstrated that MCL1 is targetable using current small molecule inhibitors, and its inhibition leads to potent anticancer activity across pediatric HGG cell lines irrespective of genotype. Employing predictive modeling approaches on a large set of childhood cancer cell lines with multiomics data features, we identified a potentially previously unreported cluster of CpG sites in the antiapoptotic BCL-xL/BCL2L1 gene, which predicted MCL1 inhibitor response. We extended these data across multiple pediatric tumor types, showing that *BCL2L1* methylation is a broad predictor of MCL1 dependency in vitro and in vivo. Overall, our multidimensional, integrated genomic approach identified MCL1 as a promising therapeutic target in several *BCL2L1*-methylated pediatric cancers, offering a translational strategy to identify patients most likely to benefit from MCL1 inhibitor therapy.

## Abstract 2

We identify BCL2L1 DNA methylation as a key biomarker predicting MCL1 dependency, offering targeted MCL1 inhibitor therapy for pediatric high-grade gliomas and other cancers.

## Graphical abstract

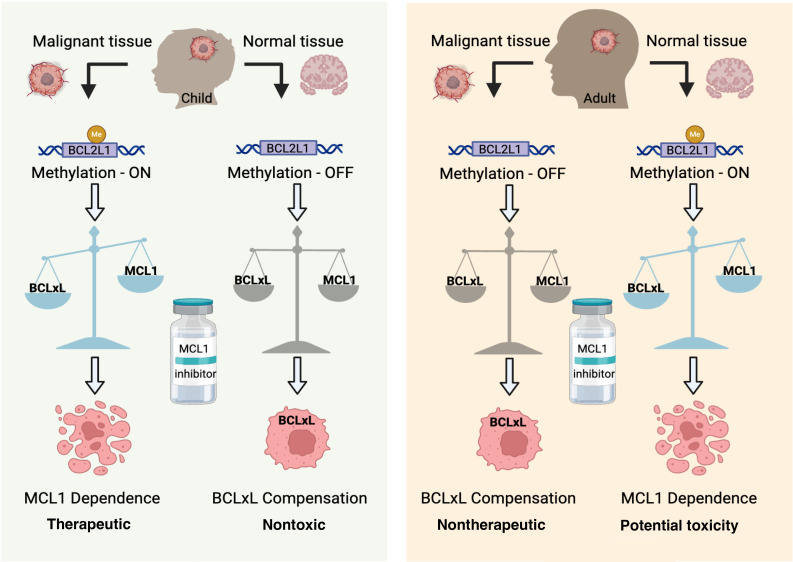

## Introduction

High-grade gliomas (HGGs) are malignant CNS neoplasms seen in both adult and pediatric populations (1, 2). Classified as grade 3 and grade 4 tumors by WHO, HGGs are characterized by hypercellularity, nuclear atypia, microvascular proliferation, and central necrosis (3–5). HGGs are more prevalent in adults, where over half (60%) of gliomas are diagnosed as HGGs compared with only approximately 10%–15% of CNS tumor diagnoses in children, where low-grade gliomas are more prevalent (6–8). Treatment consists of multimodal regimes combining surgery, radiotherapy, and chemotherapy. However, these treatments are ineffective, and less than 20% of patients survive 5 years following diagnosis (9–11). Therefore, there is an urgent unmet need to identify innovative therapies for this devastating disease and improve survival outcomes.

Recent advances in molecular, genetic, and epigenetic profiling have highlighted substantial differences in the underlying biology of adult and pediatric HGGs (12). For example, recent studies revealed the importance of epigenetic dysregulation driving oncogenesis in pediatric HGGs (pHGGs) by showing recurrent alterations in histone-coding genes *H3F3A* and *HIST1H3B/C*, genes that are essential for tumorigenesis (13). In contrast, adult HGGs (aHGGs) preferentially harbor mutations in components of receptor tyrosine kinase signaling pathways, such as *EGFR* and *PTEN* (14, 15). These observations have led to a paradigm shift toward targeted therapy, where treatments are directed against key oncogenic genes or pathways responsible for maintaining malignancy.

Given the differences in the underlying biology of adult and pediatric HGGs and the failure of targeted therapies because of the highly heterogeneous tumor profile of gliomas, it has become evident that future treatment should be tailored to the unique molecular attributes of the individual tumor of the individual patient (10, 16, 17). Therefore, current research attempts to utilize technologies that consider the unique features of a patient’s tumor, physiologic, molecular, genetic, and epigenetic, and integrate the data obtained with machine learning algorithms, to expedite the identification and development of drug targets and potential biomarkers in precision medicine–based clinical trials (18–21). These technologies merge functional approaches, such as high-throughput drug screens and pooled CRISPR/Cas9 growth screens, with multiomics features of the cancer (e.g., genome, methylome, transcriptome, deep proteome, and phospho-proteome data) to identify both the targeted therapy and its cognate predictive biomarker.

Using a multidimensional, integrative approach, the present study identified antiapoptotic protein myeloid cell leukemia 1 (MCL1) as a target enriched in pHGGs compared with aHGGs. Interestingly, we show in pediatric tumors that MCL1 dependency is not strongly predicted directly by the antiapoptotic BCL-xL/BCL2L1 gene expression. Strikingly, we uncover potentially heretofore-undescribed methylation marks, which are sufficient to predict sensitivity to MCL1 inhibitors in pediatric cancers. Finally, we validate this methylation mark in patient specimens and show that in an in vitro and in vivo setting it serves as a biomarker of MCL1 dependency/inhibitor response in additional pediatric cancer types, including atypical teratoid rhabdoid tumor (ATRT), ependymoma (EPD), and osteosarcoma (OS).

## Results

### MCL1 is an enriched gene dependency in pHGGs.

**Figure 1:**
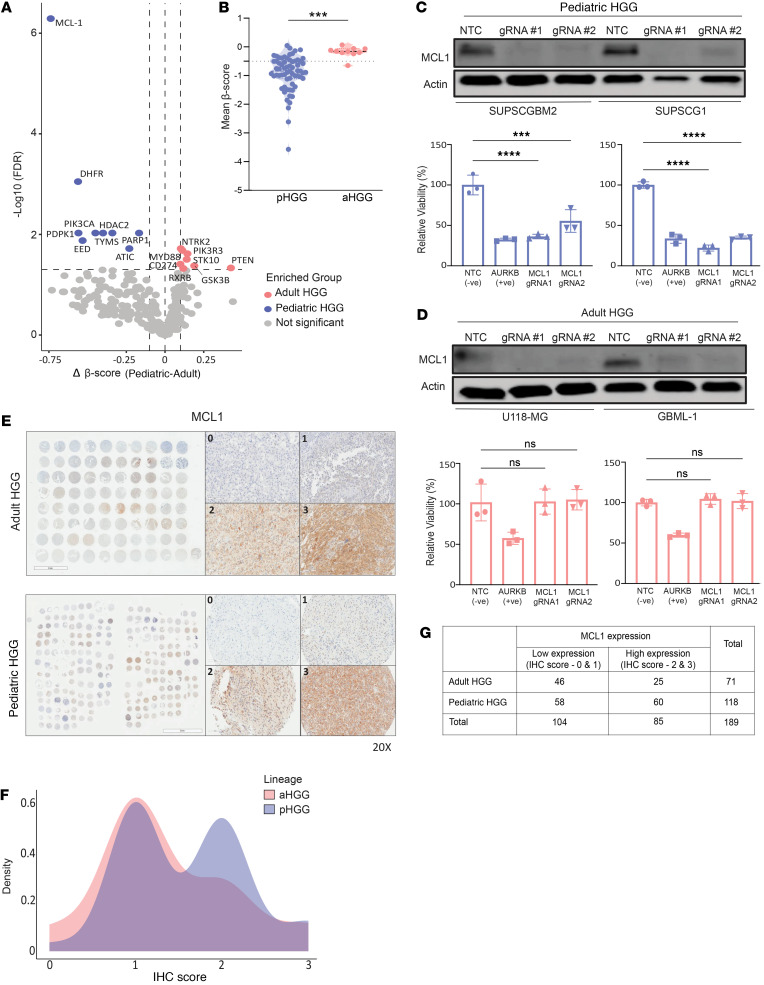
CRISPR/Cas9 screens identify MCL1 as a key genetic vulnerability in pHGG. (**A**) Relative enrichment of gene dependencies in pediatric and adult HGGs. An unpaired *t* test with multiple comparisons was used to determine statistical significance, i.e., FDR < 0.05. Δ mean β-score was calculated as mean β-score ^(Adult)^ – mean β-score ^(Pediatric)^. (**B**) Comparison of *MCL1* gene dependency scores (mean β-scores) of pediatric and adult HGG cell lines. Student’s *t* test ***P < 0.001. (**C** and **D**) Relative viability (to nontargeting control [NTC] sgRNA) of (**C**) pediatric and (**D**) adult HGG cell lines after treatment with 2 independent *MCL1*-targeting sgRNAs. Immunoblot shows MCL1 protein levels under the indicated conditions. One-way ANOVA with Bonferroni’s multiple comparisons test was used to determine significance between NTC and MCL1 sgRNAs. ***P < 0.001, ****P < 0.0001. (**E**) Representative immunohistochemical (IHC) images show MCL1-positive and -negative staining in pediatric and adult HGG tissue microarrays (TMAs) (IHC scores; 0 = null, 1 = weak, 2 = moderate, 3 = strong). Original magnification, 20×. (**F**) Density plot showing IHC score distribution of MCL1 staining in pediatric (shown in blue) and adult (pink) HGG tumor samples. (**G**) The contingency table represents the association between MCL1 expression levels (low & high) and lineage type. Fisher’s exact test.

To identify therapeutic targets distinct to pHGGs compared with their adult counterparts, we performed pooled CRISPR/Cas9 loss-of-function screens across a large cohort of adult (*n*_aHGG_ = 10) and pediatric (*n*_pHGG_ = 65) HGG cell lines (22) (Supplemental Table 1; supplemental material available online with this article; https://doi.org/10.1172/jci.insight.184601DS1). Seventeen genes were found to have age-specific growth effects, 8 of which were specific for aHGGs (*MYD88*, *PIK3R3*, *NTRK2*, *CD274*, *STK10*, *PTEN*, *GSK3B*, and *RXRB*) and 9 which were distinctly required for cellular fitness in pHGGs (*MCL1*, *DHFR*, *PDPK1*, *EED*, *PIK3CA*, *HDAC2*, *ATIC*, *PARP1*, and *TYMS*) (Figure 1A). Of these, *MCL1* was the single most enriched growth dependency in pHGGs (Supplemental Figure 1; aHGG = –0.18, pHGG = –0.92, *P* = 0.0005).

Comparison of gene dependency across the HGG cohort revealed that *MCL1* dependency was significantly more prevalent in pHGG (75%; 49 of 65) compared with aHGG cell lines (10%; 1 of 10) (Figure 1B; *P* < 0.001). Consistent with our screen data, knockout (KO) of *MCL1* by 2 independent sgRNAs led to the loss of viability in pHGG cell lines SUPSCGBM2 and SUPSCG1 (Figure 1C; *P* < 0.05). AURKB was used as a positive control.

In contrast, *MCL1* depletion had no growth effect in either aHGG line, U118-MG or GBML-1 (Figure 1D; *P* = NS), validating MCL1 as an age-related dependency in HGG.

To explore whether there are expression disparities related to MCL1 dependency in HGGs, we assessed MCL1 expression levels by IHC on a tissue microarray of adult (*n* = 71) and pediatric (*n* = 118) HGG tumors, which were scored for MCL1 expression (Figure 1E and Supplemental Table 2). IHC staining of MCL1 was categorized as absent (IHC score 0), low (IHC score 1), moderate (IHC score 2), and high (IHC score 3). Interestingly, 51% (60/118) of pHGG tumor cores exhibited moderate-high MCL1 staining (score ≥ 2) compared with adult tumors, where only 35% (25/71) of the cores expressed moderate-high levels of MCL1 (Figure 1, F and G; *P* = 0.04). In addition, comparison of MCL1 RNA expression and mean MCL1 IHC scores in pHGG cores revealed no correlation, as MCL1 RNA expression levels were consistent across the spectrum of MCL1 staining intensities (Supplemental Figure 1B; *P* = NS). Collectively, these expression data show that MCL1 is a pediatric glioma–enriched genetic dependency and underscore the need for further investigation into its role in pHGGs.

### MCL1 inhibitors AZD5991/S63845 selectively kill pHGG cell lines in vitro.

**Figure 2:**
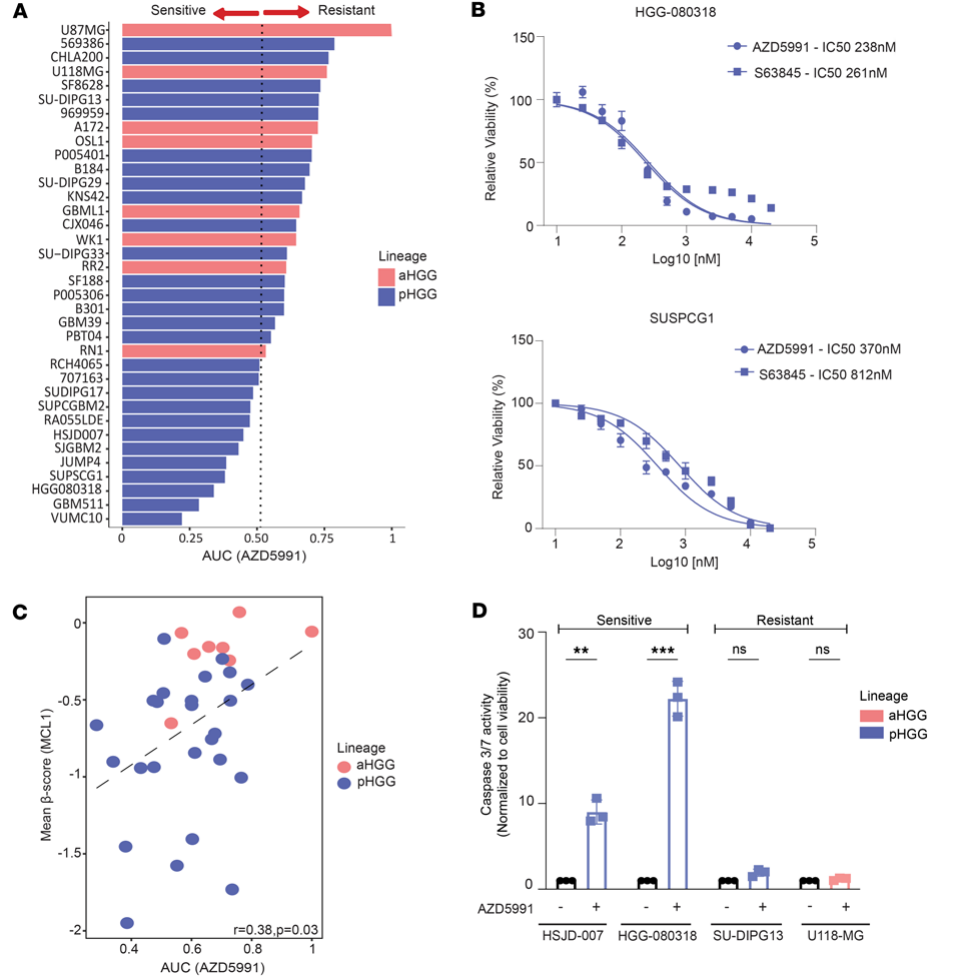
AZD5991 and S63845 show antitumor activity in HGG cell lines through a caspase-dependent pathway. (**A**) Bar plot represents AUC (normalized to U87-MG) of AZD5991 response in a panel of pediatric (*n* = 28) and adult (*n* = 8) HGG cell lines. (**B**) Drug-response curves for AZD5991 and S63845 in pHGG cell lines HGG-080318 and SUPSCG1. Representative graph (*n* ≥ 2 experiments), mean ± SEM shown. (**C**) Scatterplot showing correlation of *MCL1* gene effect (mean β-scores) of HGG cell lines (*n*^Adult^ = 8, *n*^Pediatric^ = 27) with AZD5991 drug response (AUC). Pearson’s correlation test was used to determine *r* and significance *P*. (**D**) Bar plot represents normalized caspase-3/7 activation (to control) after treatment with AZD5991 for 24 hours in sensitive and resistant cell lines. Nonparametric multiple unpaired *t* test with Bonferroni-Dunn’s test for correction of multiple comparisons was used to determine significance between – and + AZD5991. Representative graph (*n* ≥ 2 experiments), mean ± SEM shown.

Substantial progress has been made in developing MCL1 inhibitors in recent years, with many progressing to clinical trials (23). To assess the translational potential of our findings, we subjected a panel of 36 pediatric and adult HGG cell lines to 12-point dose treatments (0–20 μM) using the MCL1 inhibitor AZD5991. Consistent with our CRISPR screening data, MCL1 inhibition was broadly more effective (IC_50_ < 2 μM, AUC < 0.5) across pediatric (*n* = 12/28;43%) versus adult (*n* = 0) HGG lines (Figure 2A). Notably, a proportion of pediatric cell lines (*n* = 7/28;26%) demonstrated exquisite sensitivity to AZD5991, with antineoplastic effect observed at low nanomolar concentrations similar to the dose reported in hematological malignancies for which these compounds have progressed to clinical trials (24). In contrast, response to AZD5991 was observed only at concentrations greater than 2 μM in aHGGs (Figure 2A; AUC > 0.5). In addition, MCL1-inhibitory effects in HGG cells were investigated using an independent MCL1 inhibitor, S63845 (25). S63845 (IC_50_; HGG-080318 = 261 nM, SUPSCG1 = 812 nM) treatment showed a similar activity to AZD5991 (IC_50_; HGG-080318 = 238 nM, SUPSCG1 = 370 nM) in HGG lines, affirming the targeted effectiveness of this class of inhibitors (Figure 2B and Supplemental Figure 2A).

Correlation of gene dependence to drug activity is critical in providing insights into the mechanism of action. Comparing *MCL1* gene dependency scores with MCL1 inhibitors AZD5991 (Figure 2C; *r* = 0.38, *P* = 0.03) and S63845 (Supplemental Figure 2B; *r* = 0.64, *P* = 0.019), drug effects revealed a modest, yet statistically significant, correlation. As MCL1 has been linked to nonapoptotic functions, such as fatty acid (FA) oxidation, and as *MCL1* ablation has been reported to inhibit FA metabolism in nonmalignant tissue (26, 27), we examined the correlation between *MCL1* dependency and FA metabolism genes in pHGGs. Among the 8 FA metabolism genes identified in our CRISPR screen, none were correlated with MCL1 dependency (Supplemental Figure 2, C and D). Assessment of caspase-mediated cell death verified that MCL1 inhibition led to increased caspase-3/7 cleavage and apoptosis (*P* < 0.001), an effect not observed in cell lines resistant to AZD5991 (*P* = NS) (Figure 2D).

These findings collectively indicate that MCL1 inhibitors exhibit potent effects on pHGG, emphasizing the need for further investigation to identify biomarkers predictive of MCL1 inhibitor response in pHGG.

### A cluster of CpG methylation sites predicts MCL1 inhibitor response in pHGGs.

**Figure 3:**
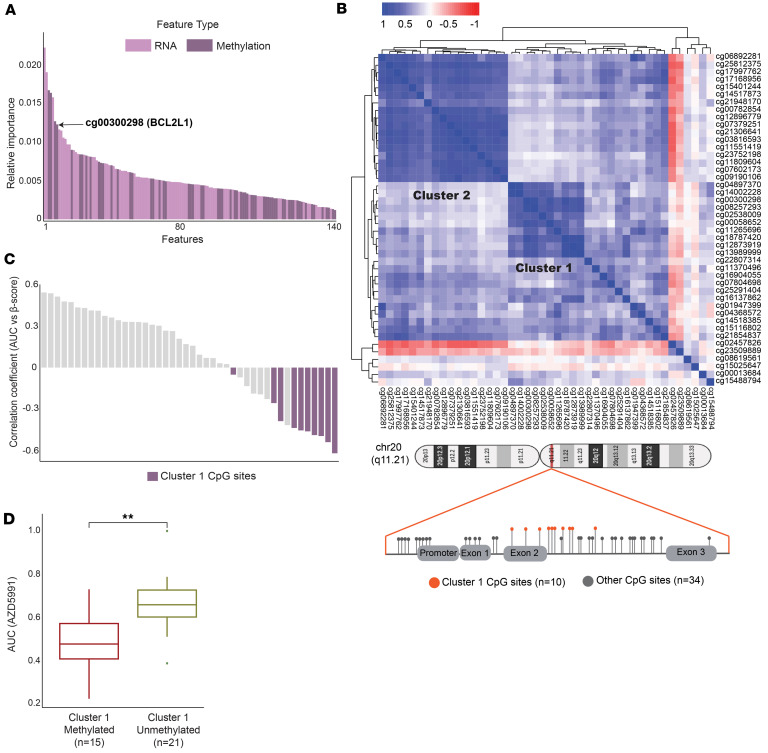
cg00300298 methylation is a potential biomarker of MCL1 inhibitor response in pHGGs. (**A**) Ranked bar plot showing predictive modeling of AZD5991 response in pHGGs. The top predictive features (predictive strength score > 0.3) of AZD5991 response plotted by the mean importance of the feature to the RF model. Features are color coded (as indicated) based on multiomics datasets, i.e., transcriptomics, and methyl omics (black, BCL2L1 methylation site cg00300298). (**B**) Hierarchical clustering of correlation coefficients of the 44 CpG sites mapping to the BCL2L1 locus. Schematic diagram, where location of cluster 1 CpG sites is color coded (*n* = 238 cell lines). (**C**) Waterfall plot showing correlation coefficient of AUC (AZD5991) versus β-score (methylation) of the 44 BCL2L1 CpG sites. Purple, cluster 1 CpG sites; gray, other CpG sites. (**D**) Box plot showing AZD5991 response of hypermethylated (*n* = 15; β-score ≥ 0.5) versus hypomethylated (*n* = 21; β-score < 0.5) cell lines at cluster 1 CpG sites. Box plots show the interquartile range, median (line), and minimum and maximum (whiskers). Student’s *t* test **P < 0.01.

We employed an unbiased artificial intelligence–based approach to identify biomarkers associated with MCL1 inhibitor response in pHGGs. Random forest (RF) machine learning models (28) were used to predict AZD5991 drug response in pHGG using RNA expression (top 8,000 variable genes), DNA methylation (top 8,000 variable CpG sites), DNA point mutations, copy number variations (CNVs), and clinical information (cancer types, sex, and age group) as input features. Model performance was evaluated by comparing the predicted drug response with the actual response in pHGG cell lines. Features were ranked according to their computed importance score, reflecting each feature’s contribution to the predictive performance of the model. Remarkably, among the 140 predictive features identified, one of the top features associated with MCL1 inhibitor (AZD5991) response in pHGGs was a potentially previously undescribed methylation site, cg00300298 within gene *BCL2L1* (Figure 3A and Supplemental Table 3). Single-correlate analysis conducted between the predictive features and MCL1 inhibitor activity (AUC of AZD5991) found that cg00300298 was ranked as the fourth most highly associated feature of AZD5991 response in pHGGs (Supplemental Figure 3A). We then examined the methylation status of 44 CpG sites mapping to the BCL2L1 locus in the Childhood Cancer Model Atlas cohort of 238 cell lines, which were subjected to Infinium methylation array (Illumina). Strikingly, hierarchical clustering of the 44 BCL2L1-associated methylation sites identified a cluster of 10 CpG sites (cluster 1), including cg00300298, that were tightly correlated (Figure 3B). An additional cluster of sites (cluster 2) corresponded to sites that were uniformly lowly methylated and were not further investigated (Supplemental Table 4). Interestingly, cluster 1 sites mapped at the exon 2 and intron 2 junction of the BCL2L1 locus and upstream of the apoptotic BCL2L1-antisense long noncoding RNA (ABALON), a known regulator of BCL2L1 isoform expression (29) (Figure 3B; http://genome.ucsc.edu). To validate these findings, single-correlate analysis was conducted between the methylation scores of the 44 BCL2L1 CpG sites and MCL1 inhibitor activity (AUC of AZD5991) across our cohort of pHGGs. Indeed, cluster 1 CpG sites mapped as the most highly correlated features. Notably, cg00300298 ranked second with a significant correlation coefficient (*r*) score –0.54 and *P* = 0.00071 (Figure 3C and Supplemental Figure 3B). Consistently, defining our cell line cohort as methylated versus unmethylated (β-score cutoff ≥ 0.5), we observed a significant association between cluster 1 methylation and MCL1 inhibitor activity (Figure 3D; *P* = 0.0013). Collectively, these results identify methylation within the *BCL2L1* gene locus (cluster 1 CpG sites) as a predictive marker for MCL1 inhibitor response in pHGGs, emphasizing the importance of further exploring its role as a biomarker in these tumors.

### cg00300298 site within BCL2L1 is hypermethylated in pediatric CNS tumors.

**Figure 4:**
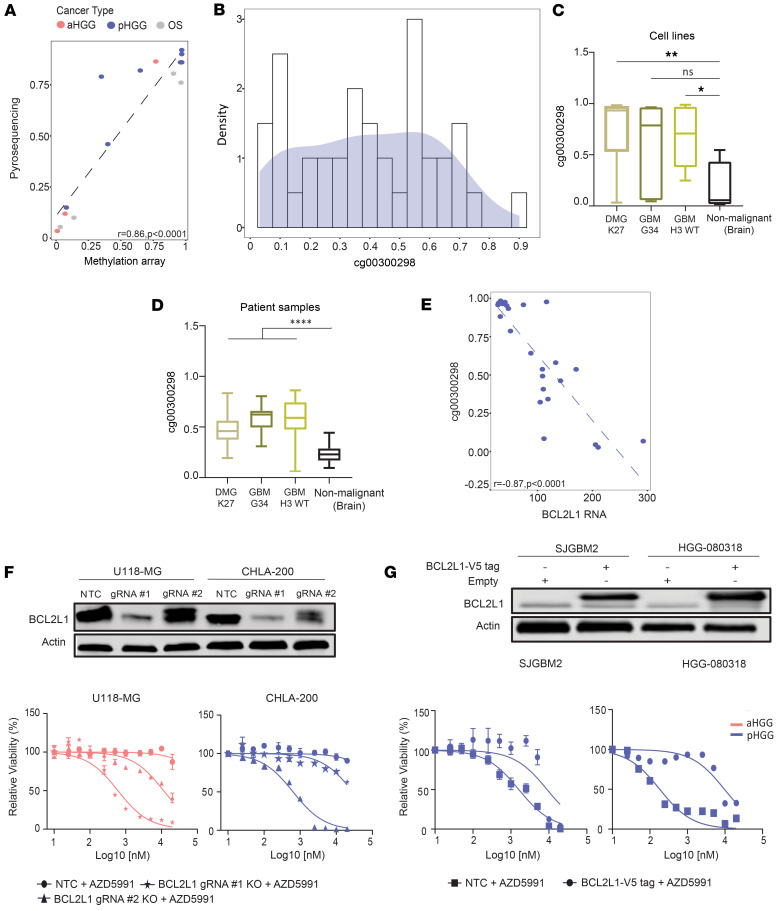
*BCL2L1* is hypermethylated in pediatric CNS tumors. (**A**) Scatterplot showing correlation of methylation array β-scores plotted by pyrosequencing values of cell lines. Data points are color coded (as indicated) based on lineage. Pearson’s correlation test was used to determine *r* and significance *P*; *P* < 0.05. (**B**) Density histogram plot showing distribution of methylation status of cg00300298 obtained via pyrosequencing across fresh patient tissue of pHGGs. (**C** and **D**) Box plots for (**C**) cell lines and (**D**) patient samples between cg00300298 methylation status of HGG subtypes and nonmalignant brain. Box plots show the interquartile range, median (line), and minimum and maximum (whiskers). One-way ANOVA with Bonferroni’s multiple comparisons test was used to determine significance between the nonmalignant cell lines/tissue and HGG groups. *P < 0.05, **P < 0.01, ****P < 0.0001. (**E**) Scatterplot showing correlation of cg00300298 methylation status plotted by *BCL2L1* RNA in pHGG. Pearson’s correlation test was used to determine *r* and significance *P*; *P* < 0.05. (**F**) Drug-response curves for AZD5991 following *BCL2L1* KO by 2 independent sgRNAs and control (NTC) in indicated cell lines. Representative graph shown (*n* = 3). Mean ± SD. Immunoblot shows BCL2L1 protein levels under indicated conditions. (**G**) Drug-response curves for AZD5991 assessed 2 weeks after sgRNA transduction of exogenous BCL2L1 and control (empty vector) in indicated cell lines. Representative graph shown (*n* = 3). Mean ± SD. Immunoblot shows BCL2L1 protein levels under indicated conditions.

Utilizing cg00300298 as an exemplar CpG site within the cluster, we first validated its methylation status by pyrosequencing across the spectrum of HGG cell lines. Pyrosequencing-derived methylation scores strongly correlated with Infinium methylation array readings and verified the predictive value of cg00300298 methylation state for MCL1 inhibitors (Figure 4A; *r* = 0.86; *P* < 0.0001). We subsequently analyzed methylation across a cohort of pHGG (*n* = 72) patient tissue samples, from which 40 (55%) passed quality control (Supplemental Figure 4A and Supplemental Table 5). An even distribution of methylation at cg00300298 was observed across the cohort of pHGG samples (Figure 4B). Comparison of pHGG with nonmalignant tissue specimens showed cg00300298 hypermethylation, verifying that *BCL2L1* was hypermethylated in both pHGG cell lines (Figure 4C; *P* = 0.005) and tumor samples (Figure 4D; *P* < 0.0001). Interestingly, cg00300298 methylation was similar across all 3 histone-altered pHGG subtypes, indicating that MCL1 dependency is not a subtype-specific vulnerability, but instead, it is a common phenotype across a spectrum of WHO-defined childhood gliomas.

Expanding our analysis to aHGG, we investigated whether methylation differences underline the observed ubiquitous lack of dependency on MCL1. Surprisingly, both adult and pediatric HGGs showed similar methylation status at cg00300298 in cell lines (Supplemental Figure 4B; *P* = NS). This was broadly recapitulated in patient tumors, where adult gliomas harbored similar methylation at the cg00300298 site compared to childhood gliomas (Supplemental Figure 4C; *P* = NS). Interestingly, deletion of *MCL1* in hypermethylated adult tumors (*n* = 5; 50%) was ineffective (Supplemental Figure 4D; *r* = 0.27, *P* = NS), consistent with the inability of cg00300298 methylation to inform MCL1 dependency in aHGGs. Together, this suggests that cg00300298 methylation is a predictor of MCL1 dependency specifically in childhood glioma and emphasizes the importance of biomarker discovery in age-relevant tumor cohorts.

Next, we explored the associations between cg00300298 methylation status and expression from proximally located genes to derive insight into how it may functionally contribute to determining MCL1 dependency. Two loci of note were identified, which could feasibly represent *cis*-regulated gene products of cg00300298: *BCL2L1* and *ABALON*. *ABALON* is a long noncoding RNA generated by alternative splicing of the primary BCL-X RNA transcript (29). The cg00300298 locus is –354 bp upstream of the ABALON transcription start site. A poor correlation was observed between cg00300298 methylation and *ABALON* mRNA expression (Supplemental Figure 4E; *P* = NS). We also evaluated expression of *BCL2L1* itself in the context of cg00300298 methylation. We found that there was a strong inverse correlation between cg00300298 methylation and *BCL2L1* RNA levels (Figure 4E; *r* = –0.87, *P* < 0.0001). Interestingly, when examining protein levels, this relationship between cg00300298 methylation and protein expression was lost (Supplemental Figure 4F; *r* = –0.51, *P* = 0.09), suggesting that BCL2-XL protein (*BCL2L1* gene product) expression, on its own, is a poor biomarker for MCL1 inhibitor sensitivity. The lack of correlation between protein and RNA is consistent with what has been described in the literature; this likely reflects the multitude of posttranscriptional and posttranslational regulatory influences that contribute to balancing apoptotic protein expression and cell death pathways (30, 31).

Given the involvement of BCL2L1 in driving apoptotic response, we then investigated whether BCL2L1 functionally contributed to determining MCL1 inhibitor response. Consistent with prior evidence, KO of *BCL2L1* led to a concentration-dependent sensitization of both adult and pediatric gliomas toward MCL1 inhibitors. Similarly, overexpression of exogenous BCL2L1 led to a reciprocal increase in resistance to MCL1 inhibition of pHGG cell lines, verifying the ability of BCL2L1 to compensate for MCL1 loss (Figure 4, F and G). Together, these data highlight that methylation of *BCL2L1* at cluster 1 CpG sites is a potential biomarker to predict MCL1 inhibitor response.

### BCL2L1 methylation is a predictor of MCL1 inhibitor response in CNS and non-CNS pediatric cancers.

**Figure 5:**
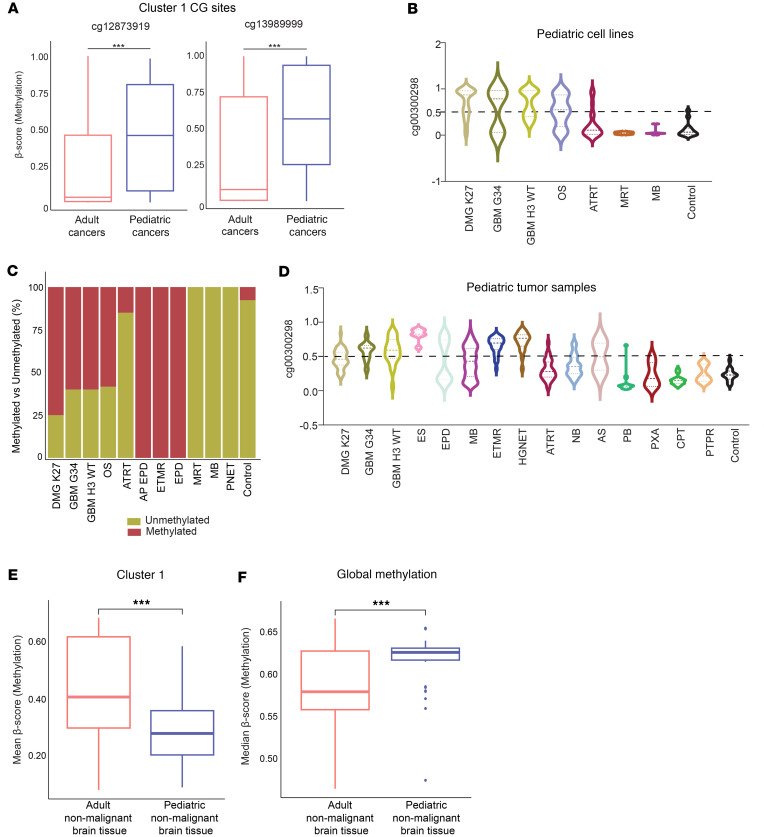
Cluster 1 methylation is a predictor of MCL1 inhibitor response in pediatric CNS and non-CNS cancers. (**A**) Box plots illustrating the distribution of cluster 1 CpG sites’ (cg12873919 and cg13989999) methylation status across pediatric and adult cancers, derived from DepMap datasets. ***P < 0.0001. (**B**) Violin plots depicting the distribution of cg00300298 methylation status across various pediatric cancers and nonmalignant brain cell lines. (**C**) Stacked bar plot represents the percentage of methylated versus unmethylated pediatric cancer cell lines based on a β-score > 0.5. (**D**) Violin plots depicting the distribution of cg00300298 methylation status across various pediatric cancers and nonmalignant brain patient tissue samples. (**E**) Box plots illustrating the mean distribution of cluster 1 CpG sites’ methylation status across nonmalignant pediatric (*n* = 36) and adult (*n* = 182) brain tissue, derived from an external dataset (35). Age cutoff to define adult and pediatric = 21, Student’s *t* test ***P < 0.0001. (**F**) Box plots illustrating the median global methylation patterns across nonmalignant pediatric (*n* = 36) and adult (*n* = 182) brain tissue, derived from an external dataset (35). Box plots show the interquartile range, median (line), and minimum and maximum (whiskers). Age cutoff = 21, Student’s *t* test ***P < 0.0001.

We next interrogated the methylation of cluster 1 CpG sites as a predictive biomarker for MCL1 inhibition in other CNS and non-CNS cancers. Interestingly, analysis of the Cancer Dependency Map (DepMap) cell lines by the Broad Institute identified 2 CpG sites of cluster 1, cg12873919 and cg13989999, which were significantly methylated in pediatric cancers compared with adult counterparts (Figure 5A; *P* < 0.001).

Evaluation of the Childhood Cancer Model Atlas cell lines revealed a continuum of methylation levels at cg00300298 with OS (*n* = 12) and ATRT (*n* = 20) showing hypermethylation in a proportion of samples. In contrast, malignant rhabdoid tumor (MRT; *n* = 3), medulloblastoma (MB; *n* = 7), and nonmalignant brain cell lines (control; *n* = 13) were uniformly hypomethylated at the cg00300298 mark (Figure 5B). We then defined a methylation (β-score) threshold by evaluating AZD5991 response and several cg00300298 methylation cutoffs using Fisher’s exact testing (Supplemental Figure 5A). Using a low-confidence cg00300298 β-score cutoff of ≥0.5 to define hypermethylation, we then binned cell lines into cg00300298 hypermethylated (β-score > 0.5) and hypomethylated (β-score < 0.5). cg00300298 hypermethylation was found to be most accentuated in anaplastic ependymoma (AP EPD; *n* = 1/1), embryonal tumors with multilayered rosettes (ETMR; *n* = 1/1), EPD (*n* = 1/1), OS (*n* = 7/12), and pHGG cell lines (42/63). In contrast, embryonal type pediatric tumors, including ATRT (17/20), MRT (*n* = 3/3), MB (*n* = 7/7), and primitive neuroectodermal tumor (PNET; *n* = 1/1), were marked by cg00300298 hypomethylation (Figure 5C). Examination of patient tumor data from German Cancer Research Center (DFKZ) datasets showed lower overall methylation levels compared with cell lines, consistent with low tumor purity of patient specimens, which likely have varying proportions of largely hypomethylated nontumor cells (32).

Generally, Ewing sarcoma (ES; *n* = 7), ETMR (*n* = 30), and high-grade neuroepithelial tumor (HGNET; *n* = 21) exhibited higher methylation levels at cg00300298 relative to other cancer types or nonmalignant tissue. Conversely, ATRT (*n* = 92), neuroblastoma (NB; *n* = 38), astrocytoma (AS; *n* = 2), pineoblastoma (PB; *n* = 16), pleomorphic xanthoastrocytoma (PXA; *n* = 6), choroid plexus tumor (CPT; *n* = 25), papillary tumor of the pineal region (PTPR; *n* = 13), and nonmalignant brain tissue (control; *n* = 16) were more hypomethylated. EPD (*n* = 126), MB (*n* = 315), and AS (*n* = 2) showed variable methylation across tumors (Figure 5D).

The relationship between global DNA methylation and age has been previously documented (33, 34), prompting us to investigate whether *BCL2L1* hypermethylation was an age-related phenomenon. We utilized DNA methylation datasets derived from normal brain across a spectrum of ages 13–96 to explore the methylation pattern of the cluster 1 CpG sites in nonmalignant CNS tissue (35). Notably, we observed an age-dependent methylation pattern across various brain regions (cerebral cortex, cerebellum, striatum, and hippocampus), with pediatric (*n* = 36) samples exhibiting a significantly hypomethylated phenotype compared with adult (*n* = 182) samples (Figure 5E; age cutoff = 21, *P* < 0.0001; Supplemental Figure 5B; and Supplemental Table 6). However, when examining global methylation patterns, the pediatric population displayed a significantly hypermethylated genome compared with the adult population (Figure 5F; *P* < 0.0001), suggesting that hypermethylation at cluster 1 CpG sites is a distinctive characteristic of pediatric CNS cancers, potentially benefiting from MCL1 inhibitor therapy. These data extend our initial observations in pHGGs and identify other childhood tumor types that harbor hypermethylation at cluster 1 CpG sites.

### BCL2L1 hypermethylation predicts MCL1 dependency in vivo.

**Figure 6:**
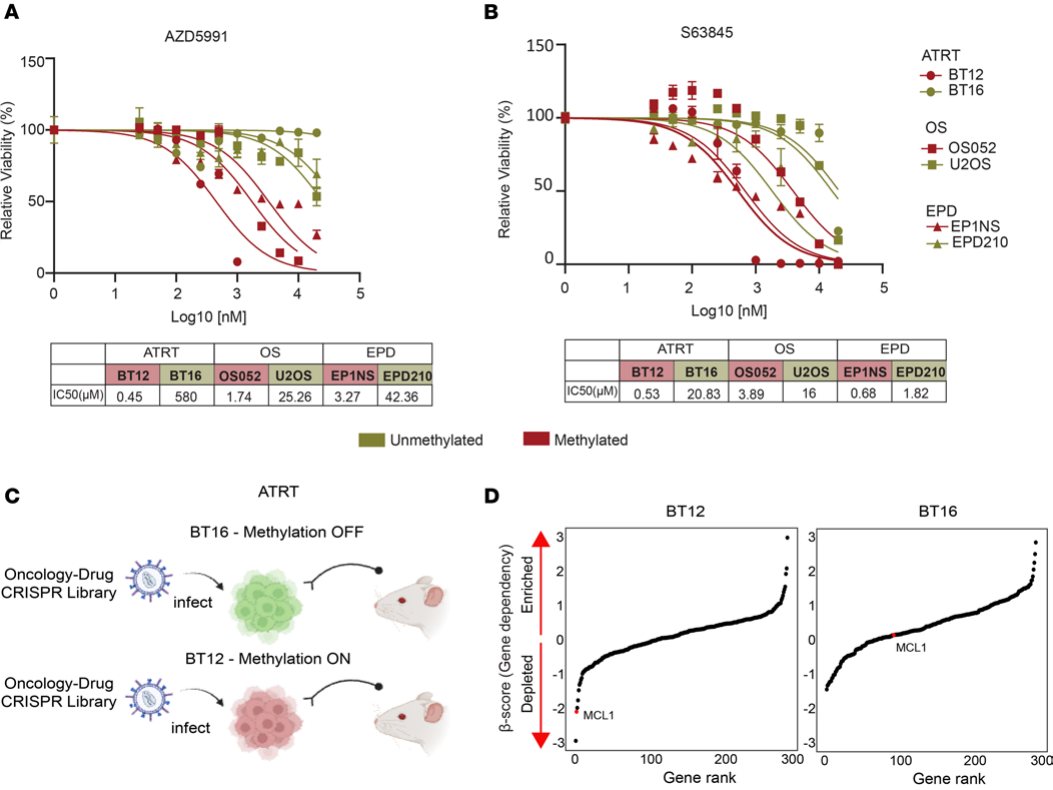
BCL2L1 hypermethylation predicts MCL1 dependency in an orthotopic CNS mouse model. (**A** and **B**) Drug-response curves for (**A**) AZD5991 and (**B**) S63845 in ATRT, OS, and EPD groups based on *BCL2L1* methylation as color coded: methylated (red) or unmethylated (green). Representative graph (*n* ≥ 2 experiments); mean ± SEM shown; extra sum-of-squares F test *P* < 0.0001. (**C**) Schematic of in vivo CRISPR/Cas9 screen utilizing orthotopic xenograft models of ATRT (created with BioRender.com). (**D**) Genes ranked by gene dependency scores (β-score) for BT12 and BT16 orthotopic in vivo screens. *MCL1* dependency color coded in red.

To define whether MCL1 inhibitor therapy in non-pHGG tumors can be defined by the cluster 1 methylation status, we tested AZD5991 and S63845 responses in selected pairs of ATRT, EPD, and OS cell lines defined by cluster 1 methylation status. Consistent with our finding in pHGG, we found that both activity of MCL1 inhibitors AZD5991 (Figure 6A, F test *P* < 0.0001) and S63845 (Figure 6B; F test *P* < 0.0001) correlated to cg00300298 methylation state. For instance, hypermethylated ATRT (BT12) responded at low nanomolar concentrations to both AZD5991 (IC_50_ = 425 nM) and S63845 (IC_50_ = 528 nM) compared with the matching hypomethylated ATRT line BT16 (IC_50_ > 20 μM for both inhibitors), representing a greater than 40-fold increase in sensitivity to MCL1 inhibitors.

We conducted in vivo pooled CRISPR/Cas9 loss-of-function screens to investigate the dependency of MCL1 in BCL2L1-hypermethylated compared with -hypomethylated models. Specifically, ATRT-BT12 (hypermethylated) and ATRT-BT16 (hypomethylated) were transduced with a pooled sgRNA library (1,666 genes/352 genes, as described above) and implanted intracranially in NOD/SCID-γ (NSG) mice 5 days after transduction (Figure 6C). Quality control analyses indicated excellent screen performance, as sgRNAs were well represented and well correlated across replicates (Supplemental Figure 6A; Pearson’s correlation > 0.5; and Supplemental Table 7). Consistent with our in vitro data, BT12 (BCL2L1 hypermethylated) was dependent on MCL1 for in vivo growth as compared with the BT16 (BCL2L1 hypomethylated). Remarkably, in BT12 tumors, MCL1 was the highest ranked gene dependency across 290 oncology-focused gene targets and showed essentiality scores on par with positive control common essential genes specifically in the presence of BCL2L1 cluster 1 hypermethylation (Figure 6D; BT12 = 2.04, BT16 = 0.21; and Supplemental Figure 6B). These findings demonstrate the use of cluster 1 methylation as a biomarker of MCL1 dependency and targeted inhibitor response is both broadly applicable and clinically tractable for a diverse set of pediatric tumors.

## Discussion

pHGGs are well known to be divergent from adults in terms of their genetic complexity, driver mutations, underlying mutational processes, and response to therapy (36). Integrating a multidimensional approach that incorporates mapping functional dependencies and molecular alterations increases our capacity to identify biomarker-coupled, targetable vulnerabilities in pHGG (22). Against this backdrop, we undertook unbiased targeted CRISPR/Cas9 loss-of-function screens targeting 364 genes to identify unique therapeutic opportunities in pHGGs. The dependencies identified in our screen included previously reported reliance on epigenetic regulation (*HDAC2*, *EED*) and the PI3K/AKT pathway (*PIK3CA*, *PDPK1*, *PTEN*, *PIK3R3*) (37–39). However, one robust and understudied dependency that was identified through our screens was the apoptosis regulator *MCL1*.

MCL1 is known to disrupt BAK/BAX/caspase-3,7–dependent apoptosis, and pharmacological inhibition by AZD5991/S63845 inhibits the binding of BAK and BAX to MCL1, resulting in cancer cell death (24, 25). Although the majority of pHGGs were susceptible to the loss of the *MCL1* gene, only a minority experienced a robust effect from MCL1 inhibition. This can be attributed to the difference in molecular techniques where one involves the complete loss of the gene *MCL1* and the other causes stabilization of the MCL1 protein via enhanced de-ubiquitination and dissociation of MCL1 from NOXA, BAK, and BAX (40).

Evasion of apoptosis is a hallmark of cancer, and therefore it is not surprising that the BCL-2 family of proteins play a key role in tumor formation and survival. BCL2L1 upregulation is a resistance factor of MCL1 inhibitor response in solid tumors and hematological malignancies (41). Although we verified that BCL2L1 does play a role in driving MCL1 inhibitor response in gliomas, our approach of integration of multiomics molecular and functional analyses identified an unprecedented ability of *BCL2L1* methylation at an exonic-intronic region (N-shore of CpG island) to predict MCL1 inhibitor response in pHGGs compared with aHGGs. Similar to DNA methylation changes at promoter and CpG islands, nonisland regions such as shores are known to regulate gene expression in cancer (42–44), consistent with the correlation we found between the cg00300298 mark and BCL2L1 expression. The BCL-2 family of proteins undergo phosphorylation, ubiquitination, proteolytic cleavage, and proteasomal degradation (45, 46), underscoring the importance of using binary marks such as DNA methylation as opposed to relying on protein and RNA expression alone. Similar to epigenetic silencing of *BCL2L1*, RNA expression exhibited a robust association with MCL1 inhibitor response. However, as methylation of DNA is considered a stable change in DNA that is less susceptible to treatment-induced alterations compared with gene expression, *BCL2L1* methylation at cg00300298 may be a better predictor of MCL1 inhibitor response in pediatric cancer (47, 48).

While the study provided valuable insights, certain limitations should be acknowledged. Comprehensive studies are needed to elucidate whether *BCL2L1* methylation at cluster 1 CpG sites directly regulates BCL2L1 itself or if it influences other factors beyond BCL2L1. Several MCL1 inhibitors are currently in clinical trials (ClinicalTrials.gov NCT05209152, NCT03672695, NCT02979366, NCT04178902) (49), but their impact on cardiac function raises safety concerns. Given that both MCL1 and BCL2L1 provide duplicate safeguard measures conferring the survival of cardiomyocytes, there is optimism that MCL1 inhibitors like ANJ810 and TTX-180 may offer clinically feasible options, as preclinical models have shown no evidence of cardiac toxicity with these inhibitors (50, 51). However, we cannot be entirely certain that such toxicity will not emerge in future clinical trials, as preclinical models may not fully predict human cardiac responses. Future studies should also focus on using MCL1 inhibitors in combination with other targeted therapies, which allow lower doses, reducing the risk of toxicity while maintaining efficacy (49). Furthermore, the capacity of existing MCL1 inhibitors to cross the blood-brain barrier and reach therapeutic concentrations within the CNS remains a major challenge (52). Consequently, future MCL1 drug development efforts should prioritize overcoming these obstacles. In the immediate term, studies should focus on trialing MCL1 inhibitors in extracranial tumors, as this approach may be more feasible.

Overall, this study identifies a vulnerability to MCL1 in pHGGs and provides a broadly applicable, predictive biomarker for MCL1 inhibitor response in other pediatric malignancies. In summary, we demonstrated that the antiapoptotic protein MCL1 is essential for tumorigenesis of pHGGs and that *BCL2L1* methylation acts as a potent pantumor predictor of AZD5991/S63845 response in several pediatric cancers, including but not limited to pHGGs.

## Methods

### Sex as a biological variable.

Our study examined male and female animals, and similar findings are reported for both sexes.

### Mouse strains.

All animal experiments utilized 6- to 10-week-old NOD.CgPrkdc^scid^IL2rg^tm1wjl^/SzJ (NSG) mice. The mice were sourced from Australian Bio Resources, New South Wales, Australia. Mouse colonies were bred and maintained in-house at the Hudson Institute of Medical Research Animal Facility (Clayton, Victoria, Australia) under specific pathogen–free conditions.

### Cell lines and culture conditions.

The list of cell lines utilized in the study, along with their growth conditions, is provided in Table S1 of the first referenced study (22, 53, 54). The source of cell lines can be accessed at Childhood Cancer Model Atlas (vicpcc.org.au/dashboard). Bryan Day of QIMR Berghofer (Brisbane, Queensland, Australia) and Brett Stringer of Flinders University (Adelaide, South Australia, Australia) provided the following cell lines: RR2 and WK1 (53, 54). The KNS-42 cell line (Cell Bank Australia) has been described before (55). Fred Hutchinson Cancer Research Center provided the following cell lines: EPD-210FHTC, GBM-511FHTC, and PBT-04FHTC. The cell lines HEK293T, RN1, U118-MG, U2OS, U87-MG, and A-172 were obtained from the American Type Culture Collection. Cell lines SU-DIPG 13, SU-DIPG 29, SU-DIPG 33, SUPSCG1 (SU-pSCG-1), and SUPSCGBM2 (SU-pcGBM2) were from Michelle Monje, Stanford University, Stanford, California, USA. Chris Jones of the Institute of Cancer Research (ICR) provided the following cell lines: ICR-B184-2D, ICR-B301-2D, and ICR-CXJ-046. Children’s Oncology group provided the following cell line: CHLA200.

In brief, to obtain single-cell suspensions, suspension cell lines were collected and centrifuged at 300*g* for 5 minutes to remove supernatant. Accutase (Thermo Fisher Scientific, Life Technologies) was added to the cell pellet, mixed, and incubated at 37°C at 5% CO_2_ for approximately 5 minutes. Medium was added to inactivate Accutase, centrifuged at 300*g* to remove supernatant, and then resuspended in fresh media. Adherent cell lines were washed with PBS, then incubated with TrypLE Select Enzyme (1×) (Thermo Fisher Scientific, Life Technologies) at 37°C at 5% CO_2_ for approximately 5 minutes to allow cell detachment. Fresh medium was then added to inactivate the TrypLE Select Enzyme before centrifugation at 300*g* and resuspension in fresh media. All cells were cultured aseptically at 37°C in humidified incubators with 5% CO_2._

### Lentiviral production.

Lentivirus production for *MCL1* sgRNA, *BCL2L1* sgRNA, and BCL2L1 overexpression vector were as follows: On day 1 HEK293T cells were seeded and incubated overnight at 37°C. The following day, a mixture of 1.2 μg psPAX (Addgene; 12260), 0.6 μg pMD2 (Addgene; 12259), and 1.2 μg of vector plasmid was diluted in 500 μL of Opti-MEM (Thermo Fisher Scientific, Gibco) and 10 μL Lipofectamine 2000 (Thermo Fisher Scientific, Invitrogen) and incubated at room temperature for 30 minutes. The transfection solution was then added dropwise to HEK293T cells and incubated overnight at 37°C. The following day, medium was replaced with 30% FBS DMEM and incubated for a further 24 hours, after which virus-containing supernatant was collected at 24- and 48-hour time points. The virus was further concentrated with Lenti-X concentrator (Takara Bio), resuspended in PBS, and aliquoted and stored at –80°C.

### Virus transduction.

Lentivirus was added to cells with polybrene (2 μg/mL) (Thermo Fisher Scientific, Life Technologies) before incubation overnight at 37°C. Twenty-four hours following transduction, selection was initiated with either blasticidin (5 μg/mL) or puromycin (2 μg/mL) (Thermo Fisher Scientific, Life Technologies).

### Establishment of modified cell lines.

Two MCL1- or BCL2L1-targeted sgRNAs were synthesized (IDT DNA) and cloned into a pLenti guide puro vector (Addgene 52963) using the Golden Gate cloning method, and lentivirus was produced for each guide as described previously (56). *AURKB* sgRNA (cell-killing positive control) and nontargeting control sgRNA (negative control) were previously cloned and sequence validated. For validation experiments, Cas9 2A-Blast cells (Addgene plasmid 73310) were seeded for each condition, then transduced 24 hours later. Puromycin (2 μg/mL) selection was maintained from day 3 to day 7, when viability was assessed using an alamarBlue assay (Thermo Fisher Scientific, Gibco). BCL2L1_PLX307 (BCL2L1-overexpression vector) was a gift from William Hahn and Sefi Rosenbluh (Addgene plasmid 98323) from Monash University, Melbourne, Victoria, Australia. sgRNA sequences were (5′ to 3′) MCL1 sgRNA1 — AGGCGCTGGAGACCTTACGA, MCL1 sgRNA2 — GTAATAACACCAGTACGGAC, BCL2L1 sgRNA1 — CAGGCGACGAGTTTGAACTG, and BCL2L1 sgRNA2 — CTCCGATTCAGTCCCTTCTG.

### Western blotting.

Cells were harvested and lysed with RIPA buffer. After measuring the protein concentration using the bicinchoninic acid assay (Thermo Fisher Scientific), the required amount of protein was separated using 4%–12% SDS-PAGE gel (Bolt, Thermo Fisher Scientific, Life Technologies), then transferred to the PVDF membrane. The membrane was blocked with blocking buffer (LI-COR Biosciences), then incubated with primary Ab at 4°C overnight, followed by incubation with a secondary Ab for 1 hour before visualization (Odyssey; LI-COR Biosciences). β-Actin was used as a loading control. Primary Abs were MCL1 (32087, Abcam), BCL2L1 (2764, Cell Signaling Technology), and β-Actin (MA5-15739-D800, Thermo Fisher Scientific). Secondary Abs were goat anti-rabbit 680RD (926-68071, LI-COR Biosciences) and goat anti-rabbit 800CW (926-32211, LI-COR Biosciences).

### Targeted pooled CRISPR/Cas9 loss-of-function screens.

Targeted pooled CRISPR/Cas9 screens performed on cell lines in this study were executed according to a previous study (22).

For in vitro screens, in brief, a customized sgRNA library consisting of 1,666 sgRNAs corresponding to 352 target genes at 4 sgRNAs per gene was utilized to perform pooled KO genetic screens. The genes included 168 oncology drug targets, 66 oncology preclinical targets, 56 cancer genes, 62 core essential genes (positive controls), and 2 nontargeting negative control genes with 250 sgRNAs. To achieve a 500× sgRNA representation (1× sgRNA to infect 500 cells), sgRNAs were introduced into Cas9-expressing cell lines we generated using Cas9 2A-Blast in replicate via lentiviral transduction at a multiplicity of infection of 0.3. Positively transduced cells were selected using 2 μg/mL puromycin throughout the 21-day screen.

For in vivo screens, 2.5 million cells infected with the previously described sgRNA library, ensuring a greater than 1,000× sgRNA representation, were transplanted intracranially in NSG immunodeficient mice (5 × 10^6^ cells/mouse; *n* = 5), in duplicate. The mice were observed daily for 7 days, then thrice weekly for signs of neurological distress or 20% weight loss. At ethical endpoints, mice were euthanized, and their brains were harvested. Following the collection of cells and tumor samples, genomic DNA was obtained using the QIAGEN DNeasy Blood & Tissue kit. Samples for the 5 mice in each replicate were pooled, and the sgRNA library was amplified using a P5 forward and a uniquely barcoded P7 reverse primer (IDT), resulting in an approximately 360 bp amplicon verified by agarose gel electrophoresis. Finally, PCR products for each sample were pooled and purified using AMpure Beads (Beckman Coulter), and the amplicons along with the sgRNA library plasmid controls were submitted for next-generation sequencing.

### Caspase Glo 3/7 assay.

Following drug treatment, cells were subjected to caspase-3/7 activity measurement using the Caspase-Glo 3/7 assay kit (Promega). White-walled, 96-well plates containing cells were removed from the incubator and allowed to equilibrate to room temperature for 30 minutes. Then, 50 µL of Caspase-Glo reagent was added to each well, and the contents of the wells were gently mixed by a plate shaker at 300–500 rpm for 30 seconds. After 2 hours of incubation in room temperature, the luminescence of samples was measured with the Clario Star plate reader (BMG LABTECH). Readings were normalized to cell viability alamarBlue assay. The experiments were performed in triplicate and repeated with 3 separately initiated cultures.

### DNA extraction and bisulfite treatment.

DNA was extracted from HGG cell lines and patient tissue samples using the DNeasy Tissue Kit (QIAGEN) and subsequently bisulfite-treated using the EZ DNA Methylation Kit (Zymo Research) according to the manufacturer’s protocol to convert all unmethylated cytosine to uracil while leaving 5-methylcytosine unaltered. Tumor samples and coded data were supplied by the Children’s Cancer Centre Tissue Bank at the Murdoch Children’s Research Institute and The Royal Children’s Hospital (www.mcri.edu.au/childrenscancercentretissuebank). Tumor samples and coded data were supplied by the Monash Children’s Cancer Biobank and the Monash Children’s Hospital. ANZCHOG Biobanking Network, Sydney Children’s Tumor Bank Network (Children’s Cancer Institute Tumor Bank and Children’s Hospital at Westmead Tumor Bank) provided samples. The treated DNA was then eluted in 12 μL of M-elution buffer (Zymo Research).

### PyroMark PCR assay and pyrosequencing.

PCR and sequencing primers were designed using the PyroMark Assay Design Software. PCR assays were designed to amplify the CpG site of interest (BCL2L1: cg00300298) for subsequent pyrosequencing. HotStar PCR was carried out with the HotStar Taq Master Mix Kit (QIAGEN) using 500 ng of bisulfite-treated DNA along with a negative control. Confirmation of PCR product quality was established on a 2% agarose gel with ethidium bromide staining. Pyrosequencing was performed using the PyroMark Q48 Autoprep instrument (QIAGEN). PCR and sequencing primer sequences were (5′ to 3′) forward primer — TTTTATTTGTTTTTTTTAAGGGGTTTTAGT, reverse-Biotin primer — TCCTACCTATAACCATACCCTAATCT, sequencing primer — AAGTTTTTTTTATTTTAAAGTTTG

### Drug sensitivity assay.

AZD5991 (S8643) was purchased from Selleckchem whereas S63845 (C-1370) was purchased from Active Biochem (Assay Matrix). Drugs were dissolved in DMSO unless otherwise indicated, aliquoted, and stored at –80°C, where they were freeze-thawed up to 5 times prior to disposal. Cells were seeded in triplicate into 96-well plates, then treated at concentrations ranging 0–20 μM. Cell viability was assessed after 72 hours by an alamarBlue assay.

### IHC and imaging analysis.

IHC staining for MCL1 was performed by the Monash Histology Platform, Monash University, using the avidin-biotin-peroxidase complex method. Adult and pediatric HGG TMAs obtained from US Biomax Inc. were deparaffinized for IHC. Briefly, following deparaffinization and antigen retrieval by microwaving in EDTA buffer for 20 minutes, endogenous peroxidase activity was blocked by 0.3% H_2_O_2_ in methyl alcohol for 30 minutes. Subsequently, primary Ab MCL1 (Abcam 32087) was applied at a dilution of 1:2,000 overnight at 4°C. The sections were then incubated with a biotinylated secondary Ab (Thermo Fisher Scientific 31460) diluted 1:300 in PBS for 40 minutes followed by washing with PBS. Next, the color reaction was carried out with DAB and nuclei were counterstained with hematoxylin.

The scanned images were analyzed by Aperio ImageScope 12.3.3 software. The positivity threshold for staining was determined empirically based on controls, and the intensity was classified from 0 to 3.

### Predictive modeling of drug response using RF machine learning algorithm.

To predict biomarkers for AZD5991 sensitivity, we applied the machine learning RF method to build predictive models based on multiomics datasets (https://github.com/broadinstitute/cdsr_models; commit ID 1986dc8). In brief, for AZD5991 drug response, the top 400 correlated features were filtered to fit into a predictive model. The features included transcriptomic profiles (top 8,000 variable genes), cancer-related damaging mutations and CNVs, methylation (top 8,000 variable CpG sites), and clinical information (cancer types, sex, and age group). HGG cell lines were classified into either sensitive or resistant groups using the threshold drug sensitivity value of LogIC_50_ 3.5/IC_50_ 2 μM, and the AUC was calculated using Graph Pad Prism 9.4.1.

RF relative importance was obtained by calculating the gradient of the line of best fit between ranked importance values for each RF model. The features with the greatest contribution were obtained by dividing the gradient by the maximum importance value and the total length of features assigned to the model. Features with a derived score of > = 0.3 were considered a top contributing feature to the RF and used to normalize importance.

### Statistics.

All statistical analysis was performed using GraphPad Prism software 9.0 or R (R version 4.2.0) with appropriate tools/packages. IC_50_s of drug response were estimated using GraphPad Prism. The significant difference between 2 groups was analyzed using the unpaired Student’s 2-tailed *t* test, and for multiple-group comparisons, 1-way ANOVA with Bonferroni’s post hoc test was conducted to correct for multiple comparisons. *P* < 0.05 was considered statistically significant. Statistical tests were adjusted for multiple hypotheses’ correction using the Benjamini-Hochberg FDR with less than 5% considered significant. Correlation between variables was calculated based on the Pearson rank correlation coefficient. Quantified multiomics datasets (CRISPR dependencies, transcriptomics, and DNA methylation) were obtained (22). Genetic dependencies were determined by comparing sgRNA representation (β-score) on day 21 with day 0 plasmid reference. Genes with mean β-score ≤ 0.5 were defined as hits, signifying reduced cell viability. Unpaired *t* tests analyzed β-score across 352 genes in pediatric and adult HGG cell lines, revealing unique vulnerabilities. Hits specific to pHGG or aHGG were defined using Δ mean β-score ± 0.1, FDR < 0.05. Relevant statistical parameters are stated in the legend of each figure.

### Study approval.

All animal experiments and research plans were approved by the Monash Medical Centre ethics committee, Melbourne, Victoria, Australia (MMCA/2022/13 – Modelling Pediatric Brain Tumors in vivo).

### Data availability.

Values for all data points in graphs are reported in the Supporting Data Values file. Datasets are publicly available at vicpcc.org.au/dashboard (download tab). Whole-genome sequencing and methylation EPIC array datasets have been deposited at European Genome Archive EGA: EGAS00001006320. DKFZ methylation datasets were downloaded from Gene Expression Omnibus (GSE109381). New analytic code was not generated during this study.

## Author contributions

The studies were conceived and designed by SA, PD, and RF. Biobanking/platform services were provided by NN, DH, and LEL. Experiments were performed by SA, PD, GB, NJC, VT, HL, VGV, SG, JEC, AY, and ML. Computational analyses were performed by SA, PD, and CXS. NZ provided helpful discussions. EASC was the provider of the cell line OS052. Writing and manuscript preparation were done by SA, PD, DDE, and RF.

## Supplementary Material

Supplemental data

Unedited blot and gel images

Supplemental table 1

Supplemental table 2

Supplemental table 3

Supplemental table 4

Supplemental table 5

Supplemental table 6

Supplemental table 7

Supporting data values

## Version 1 01/23/2025

Electronic publication

## References

[1] Lapointe S (2018). Primary brain tumours in adults. Lancet.

[2] Chiang JC, Ellison DW (2017). Molecular pathology of paediatric central nervous system tumours. J Pathol.

[3] Ferris SP (2017). Characterization of gliomas: from morphology to molecules. Virchows Archiv.

[4] Louis DN (2021). The 2021 WHO classification of tumors of the central nervous system: a summary. Neuro Oncol.

[5] Hara A, Kanayama et al (2019). Treatment strategies based on histological targets against invasive and resistant glioblastoma. J Oncol.

[6] El-Ayadi M (2017). High-grade glioma in very young children: a rare and particular patient population. Oncotarget.

[7] Fangusaro J (2012). Pediatric high grade glioma: a review and update on tumor clinical characteristics and biology. Front Oncol.

[8] Hanif F (2017). Glioblastoma multiforme: a review of its epidemiology and pathogenesis through clinical presentation and treatment. Asian Pac J Cancer Prev.

[9] Sturm D (2017). Pediatric gliomas: current concepts on diagnosis, biology, and clinical management. J Clin Oncol.

[10] Williams MJ (2017). Therapeutic targeting of histone modifications in adult and pediatric high-grade glioma. Front Oncol.

[11] Sturm D (2014). Paediatric and adult glioblastoma: multiform (epi) genomic culprits emerge. Nat Rev Cancer.

[12] Jones C (2012). Paediatric and adult malignant glioma: close relatives or distant cousins?. Nat Rev Clin Oncol.

[13] Khuong-Quang DA (2012). K27M mutation in histone H3.3 defines clinically and biologically distinct subgroups of pediatric diffuse intrinsic pontine gliomas. Acta neuropathol.

[14] Rizzo D (2015). Molecular biology in pediatric high-grade glioma: impact on prognosis and treatment. Biomed Res Int.

[15] Zheng S (2019). Prospective clinical sequencing of adult glioma. Mol Cancer Ther.

[16] Patel M (2012). Molecular targeted therapy in recurrent glioblastoma: current challenges and future directions. Expert Opin InvestigDrugs.

[17] Jain KK (2018). A critical overview of targeted therapies for glioblastoma. Front Oncol.

[18] Jameson JL, Longo DL (2015). Precision medicine — personalized, problematic, and promising. N Engl J Med.

[19] Ene CI, Holland EC (2015). Personalized medicine for gliomas. Surg Neurol Int.

[20] Chakravarthi BV (2016). Genomic and epigenomic alterations in cancer. A J Pathol.

[21] Gallo Cantafio ME (2018). From single level analysis to multi-omics integrative approaches: a powerful strategy towards the precision oncology. High Throughput.

[22] Sun CX (2023). Generation and multidimensional profiling of a childhood cancer cell line atlas defines new therapeutic opportunities. Cancer Cell.

[23] Wang H (2021). Targeting MCL-1 in cancer: current status and perspectives. J Hematol Oncol.

[24] Tron AE (2018). Discovery of Mcl-1-specific inhibitor AZD5991 and preclinical activity in multiple myeloma and acute myeloid leukemia. Nature Commun.

[25] Kotschy A (2016). The MCL1 inhibitor S63845 is tolerable and effective in diverse cancer models. Nature.

[26] Wright T (2024). Anti-apoptotic MCL1 promotes long-chain fatty acid oxidation through interaction with ACSL1. Mol Cell.

[27] Prew MS (2022). MCL-1 is a master regulator of cancer dependency on fatty acid oxidation. Cell Rep.

[28] Dempster JM (2019). Gene expression has more power for predicting in vitro cancer cell vulnerabilities than genomics. J Exp Clin Cancer Res.

[29] Chen W, Li J (2021). Alternative splicing of BCL-X and implications for treating hematological malignancies. Oncol Lett.

[30] Nie L (2006). Correlation of mRNA expression and protein abundance affected by multiple sequence features related to translational efficiency in Desulfovibrio vulgaris: a quantitative analysis. Genetics.

[31] Koussounadis A (2015). Relationship between differentially expressed mRNA and mRNA-protein correlations in a xenograft model system. Sci Rep.

[32] Capper D (2018). DNA methylation-based classification of central nervous system tumours. Nature.

[33] Field AE (2018). DNA methylation clocks in aging: categories, causes, and consequences. Mol Cell.

[34] Johnson AA (2012). The role of DNA methylation in aging, rejuvenation, and age-related disease. Rejuvenation Res.

[35] Policicchio S (2020). Genome-wide DNA methylation meta-analysis in the brains of suicide completers. Transl Psychiatry.

[36] Gröbner SN (2018). The landscape of genomic alterations across childhood cancers. Nature.

[37] Velpula KK, Tsung AJ (2014). PDK1: a new therapeutic target for glioblastoma?. CNS Oncol.

[38] Zhao H-f (2017). Recent advances in the use of PI3K inhibitors for glioblastoma multiforme: current preclinical and clinical development. Mol Cancer.

[39] Vanan MI (2017). Targeting epigenetic pathways in the treatment of pediatric diffuse (high grade) gliomas. Neurotherapeutics.

[40] Tantawy SI (2023). Mechanisms of MCL1 protein stability induced by MCL-1 antagonists in B-cell malignancies. Clin Cancer Res.

[41] Yasuda Y (2020). MCL1 inhibition is effective against a subset of small-cell lung cancer with high MCL1 and low BCL-X L expression. Cell Death Dis.

[42] Rao X (2013). CpG island shore methylation regulates caveolin-1 expression in breast cancer. Oncogene.

[43] de Souza CF (2018). A distinct DNA methylation shift in a subset of glioma CpG island methylator phenotypes during tumor recurrence. Cell Rep.

[44] Zhang X (2010). Methylation of a single intronic CpG mediates expression silencing of the PMP24 gene in prostate cancer. Prostate.

[45] Roberts JZ (2022). The role of ubiquitination in apoptosis and necroptosis. Cell Death Differ.

[46] Kutuk O, Letai A (2008). Regulation of Bcl-2 family proteins by posttranslational modifications. Curr Mol Med.

[47] Karayan-Tapon L (2010). Prognostic value of O6-methylguanine-DNA methyltransferase status in glioblastoma patients, assessed by five different methods. J Neurooncol.

[48] Ding W (2019). Integrative analysis identifies potential DNA methylation biomarkers for pan-cancer diagnosis and prognosis. Epigenetics.

[49] Tantawy SI (2023). Targeting MCL-1 protein to treat cancer: opportunities and challenges. Front Oncol.

[50] Rauh U (2024). BRD-810 is a highly selective MCL1 inhibitor with optimized in vivo clearance and robust efficacy in solid and hematological tumor models. Nat Cancer.

[51] Guo L (2018). Role of Mcl-1 in regulation of cell death in human induced pluripotent stem cell-derived cardiomyocytes in vitro. Toxicol Appl Pharmacol.

[52] Houweling M (2023). Screening of predicted synergistic multi-target therapies in glioblastoma identifies new treatment strategies. Neurooncol Adv.

[53] D’Souza RC (2020). Q-Cell glioblastoma resource: proteomics analysis reveals unique cell-states are maintained in 3D culture. Cells.

[54] Stringer BW (2019). A reference collection of patient-derived cell line and xenograft models of proneural, classical and mesenchymal glioblastoma. Sci Rep.

[55] Takeshita I (1987). Characteristics of an established human glioma cell line, KNS- 42. Neurol Med Chir (Tokyo).

[56] Engler C, Marillonnet S (2014). Golden Gate cloning. Methods Mol Biol.

