# Supplementary material for: Multidimensional, integrative profiling identifies BCL2L1 methylation as a predictor of MCL1 dependency in pediatric malignancies
Source: JCI Insight. 2025 Jan 23;10(2):e184601. doi: 10.1172/jci.insight.184601 (PMC11790018; doi:10.1172/jci.insight.184601)

Supplemental Figure Legends

Supplemental Figure 1

(A) Comparison of gene dependency scores (Mean β-scores) of 8 significant genes between pediatric and adult HGG cell lines. Student t-test was performed to determine the significance of the adjusted p-value.

(B) Comparison of MCL1 RNA expression (z-score) with the mean IHC scores of pediatric HGG TMA. One-way Anova test was performed to determine the significance. Multiple comparison was corrected by using Bonferroni's test. p=ns.

Supplemental Figure 2

(A) Bar plot represents Log10 IC_{50} of AZD5991 and S63845 in a panel of pediatric and adult HGG cell lines.

(B) Scatterplot showing correlation of *MCL1* gene effect (Mean β-scores) of HGG cell lines ($n^{(Adult)}$ = 2, $n^{(Pediatric)}$ = 11) with S63845 drug response (AUC). Pearson's correlation test was used to determine r and significance p.

(C) Waterfall plot showing the negative and positive correlated fatty acid metabolism gene dependencies with *MCL1* gene dependency in pHGG. Pearson's correlation test was used to determine r and significance p.

(D) Scatterplots showing correlation between the eight-fatty acid metabolism genes with *MCL1* gene dependency in pHGG. Pearson's correlation test was used to determine r and significance p.

Supplemental Figure 3

(A) Waterfall plot showing the negative and positive correlated features with AZD5991 response in pHGG.

(B) Scatterplot showing correlation between methylation status of cluster 1 CpG sites against AZD5991 response in pediatric HGG cell lines. Pearson's correlation test was used to determine r and significance p.

Supplemental Figure 4

(A) Pyrograms of control, methylated and unmethylated cg00300298. "c" bisulphite control, "T-C" CpG site of interest.

(B-C) Box plot comparing the distribution of cg00300298 methylation status between pediatric and adult HGG subtypes in **(B)** cell lines and **(C)** patient samples. Student t-test, *$p<0.05$, ns = not significant.

(D) Scatterplot showing correlation between methylation status of cg00300298 vs Mean β-score of *MCL1* dependency in pediatric HGGs. Pearson's correlation coefficient, $p < 0.05$. Pink highlighted points indicate the methylated samples which are non-dependent on MCL1.

(E) Scatterplot showing correlation between ABALON RNA levels vs methylation status of cg00300298 in pediatric HGGs. Pearson's correlation coefficient, p =ns.

(F) Scatterplot showing correlation between BCL-xL protein levels vs methylation status of cg00300298 in pediatric HGGs. Pearson's correlation test was used to determine r and significance p, $p < 0.05$.

Supplemental Figure 5

(A) Table represents Fisher's exact test p values for methylation beta-score cut-offs ranging from 0.7-0.4 in cell lines. Student t-test. *p<0.05, **p<0.01, ***p<0.001, ****p<0.0001, ns=not significant.

(B) Boxplots illustrates the methylation patterns of the individual CpG sites (cg00300298, cg08257293, cg12873919, cg13989999, and cg18787420 across and adult and pediatric non-malignant brain tissue, derived from an external dataset (36). Student t-test. *p<0.05, **p<0.01, ***p<0.001, ****p<0.0001, ns=not significant.

Supplemental Figure 6

(A) Scatterplot showing the correlation between replicates of BT12 and BT16 in vivo genomic screens.

(B) Gene dependency scores of *MCL1* and core-essential scores of BT12 and BT16 cell lines. Red – MCL1, Grey – Core -essentials.

Supplemental Table Legends

Supplemental Table S1

In vitro CRISPR control metrics summary of adult and pediatric HGG cell lines, related to Figure 1

Supplemental Table S2

Layout and diagnosis of cores in adult and pediatric tissue microarrays (TMAs), related to Figure 1

Supplemental Table S3

Random Forest (RF) features of AZD5991 response in HGG, related to Figure 3

Supplemental Table S4

Beta-scores (Methylation scores) of the 44 BCL2L1 CpG sites, related to Figure 3

Supplemental Table S5

Clinical data of patient samples, related to Figure 4

Supplemental Table S6

Beta-scores (Methylation) of 5 overlapping cluster 1 CpG sites, related to figure 5

Supplemental Table S7

In vivo CRISPR control metrics summary of BT12 and BT16 ATRT cell lines, related to Figure 6

Supplemental Figure 1

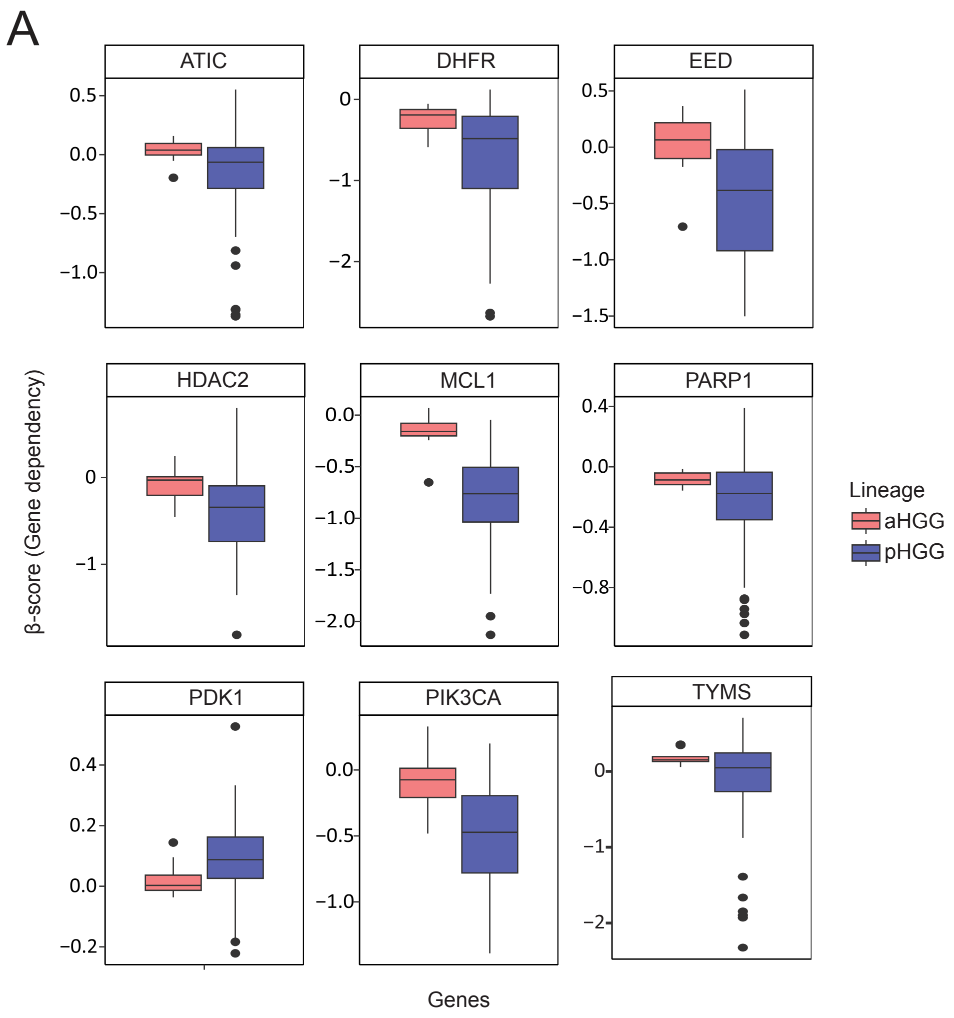

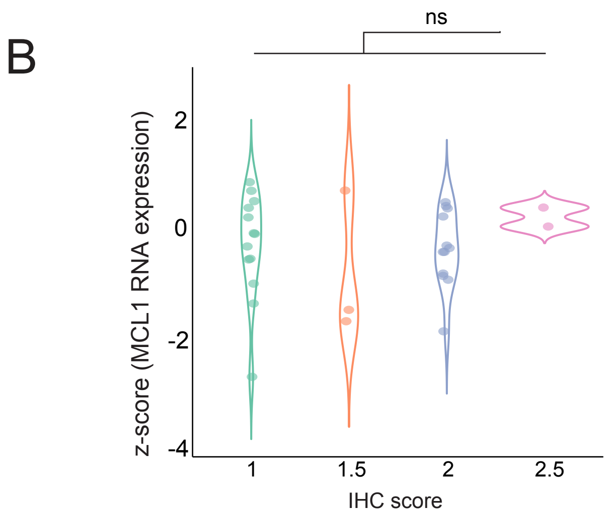

Supplemental Figure 2

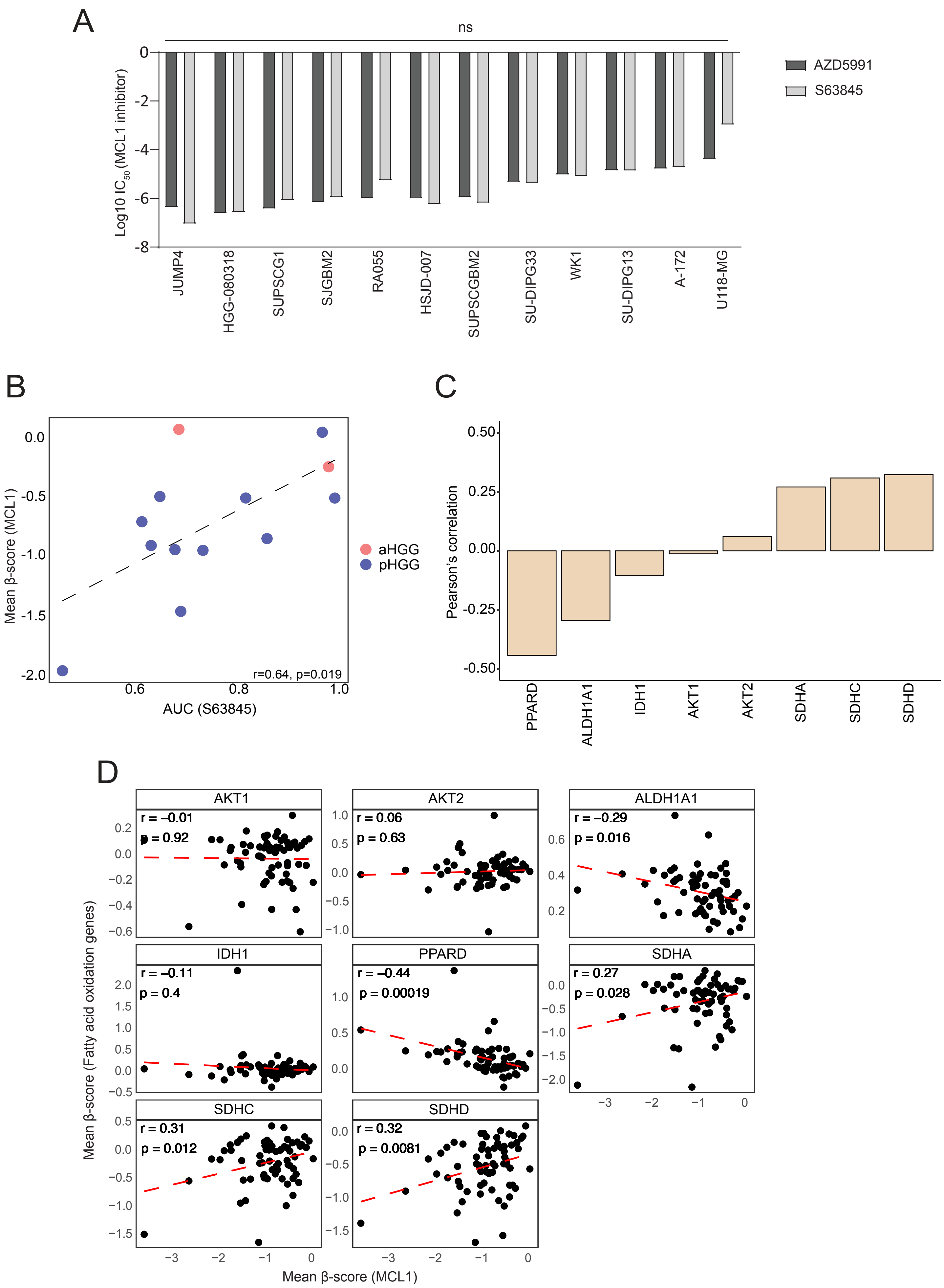

Supplemental Figure 3

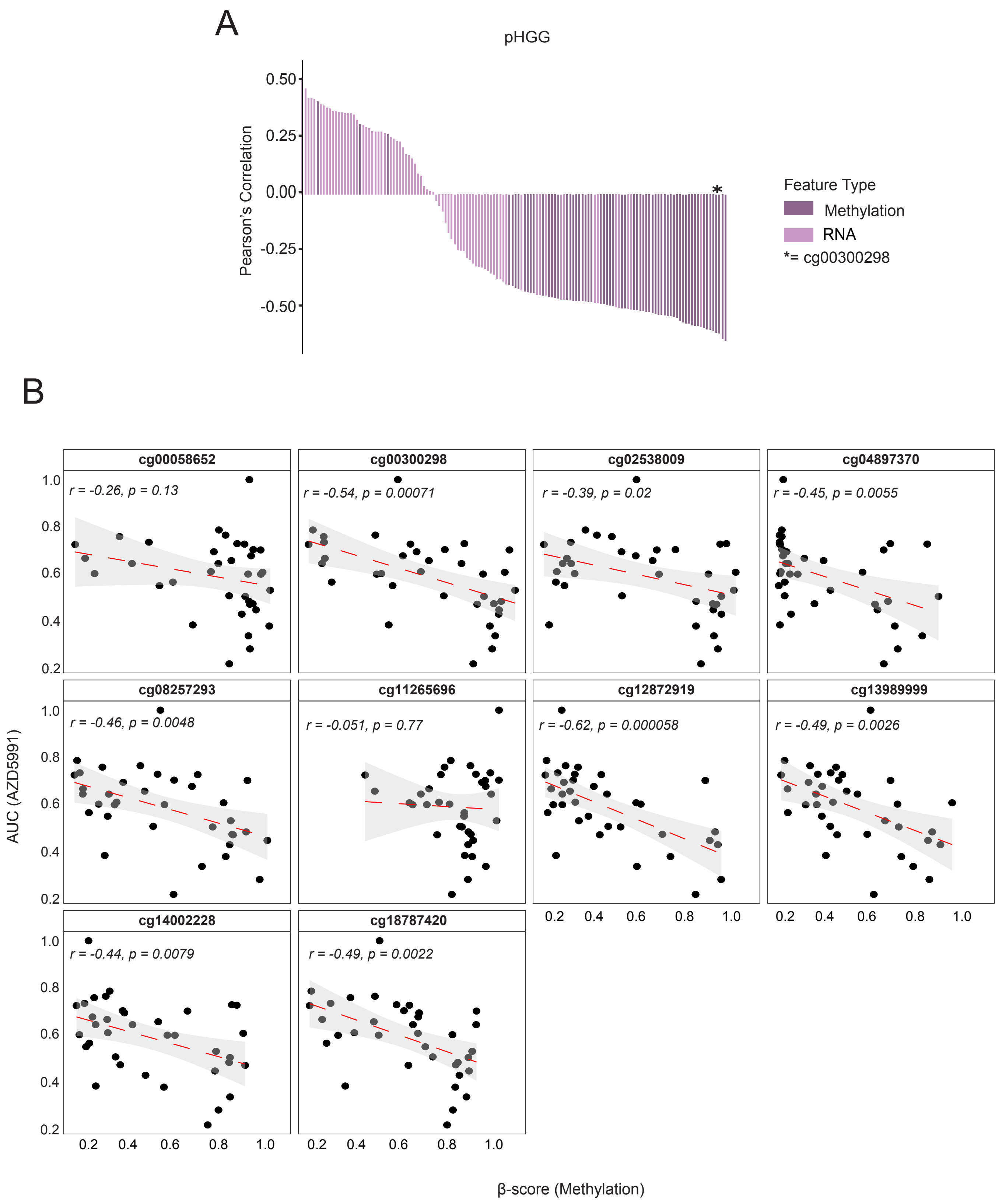

Supplemental Figure 4

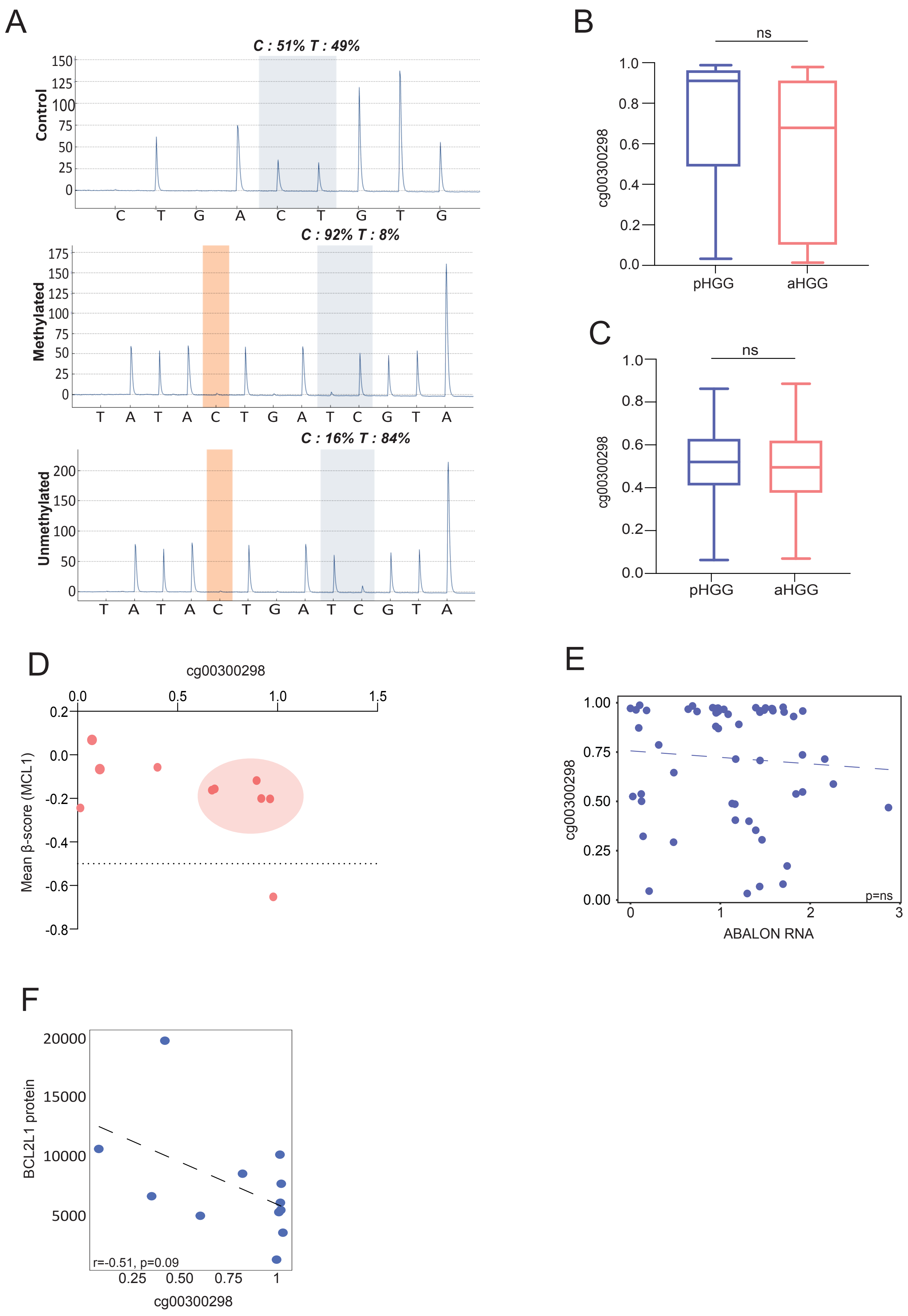

Supplemental Figure 5

A

Methylated Phenotype Threshhold (β-score)	Fisher's Exact Test (p-value)
≥0.7	***
≥0.6	**
≥0.5	*
≥0.4	ns

B

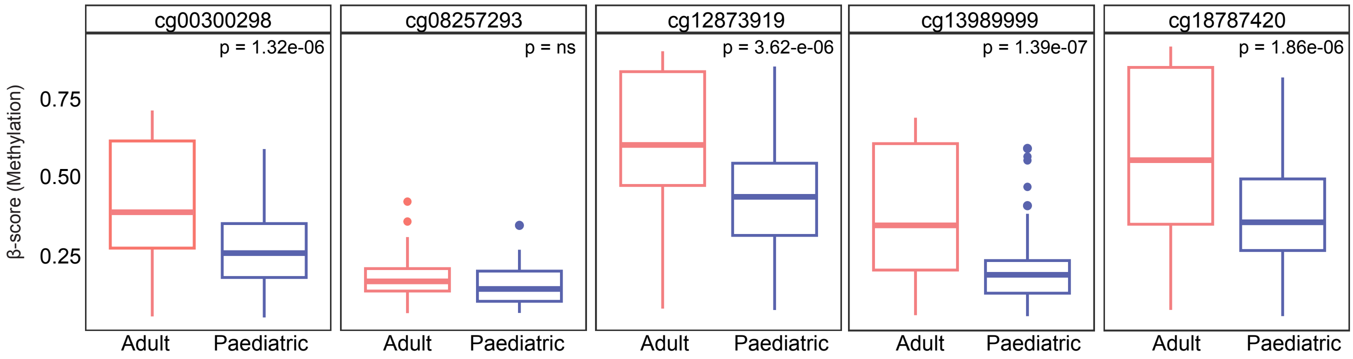

Supplemental Figure 6

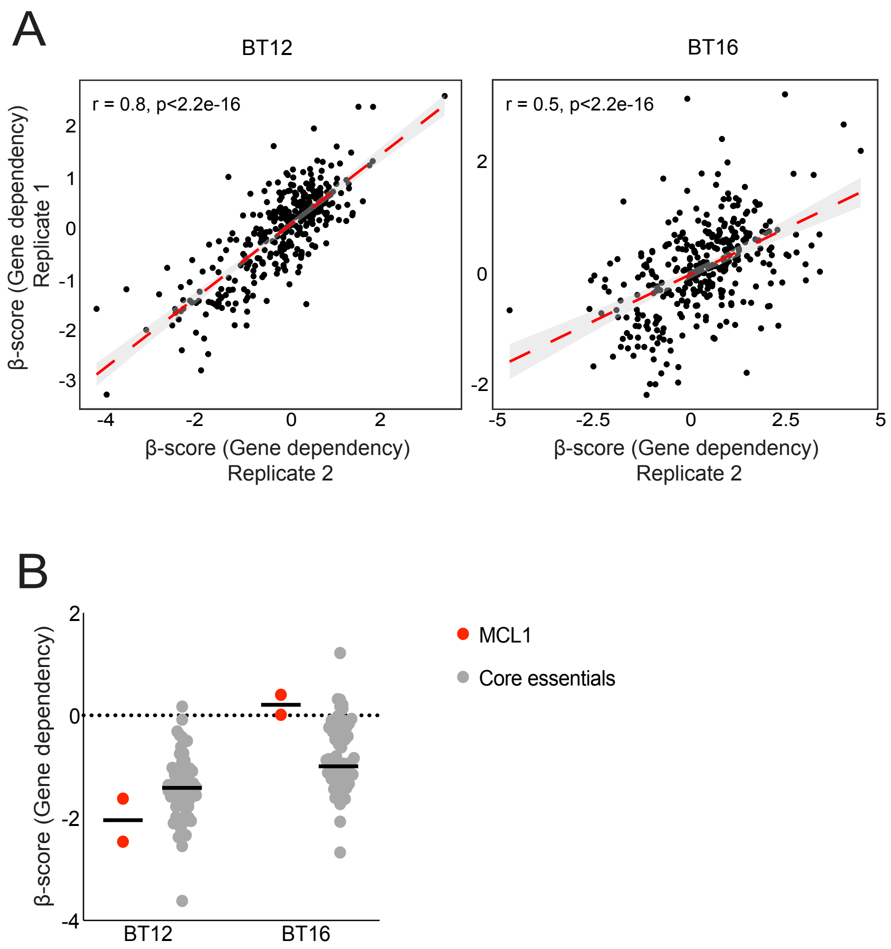

Supplement: Supplemental data [file jciinsight-10-184601-s045.pdf]
