# Supplementary figures and images for: Multidimensional, integrative profiling identifies BCL2L1 methylation as a predictor of MCL1 dependency in pediatric malignancies
Source: JCI Insight. 2025 Jan 23;10(2):e184601. doi: 10.1172/jci.insight.184601 (PMC11790018; doi:10.1172/jci.insight.184601)

Figure 1C & 1D

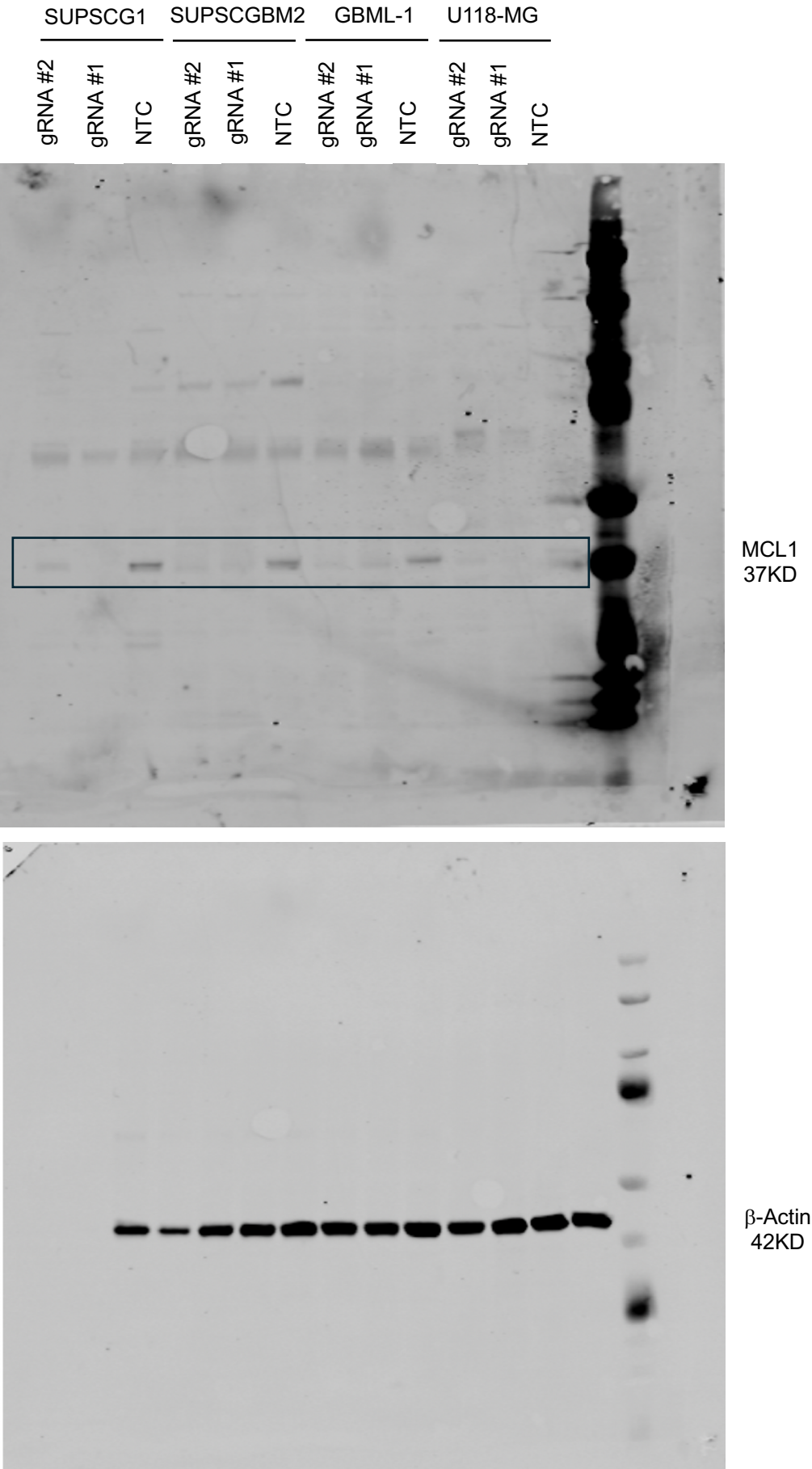

**Figure 4F**

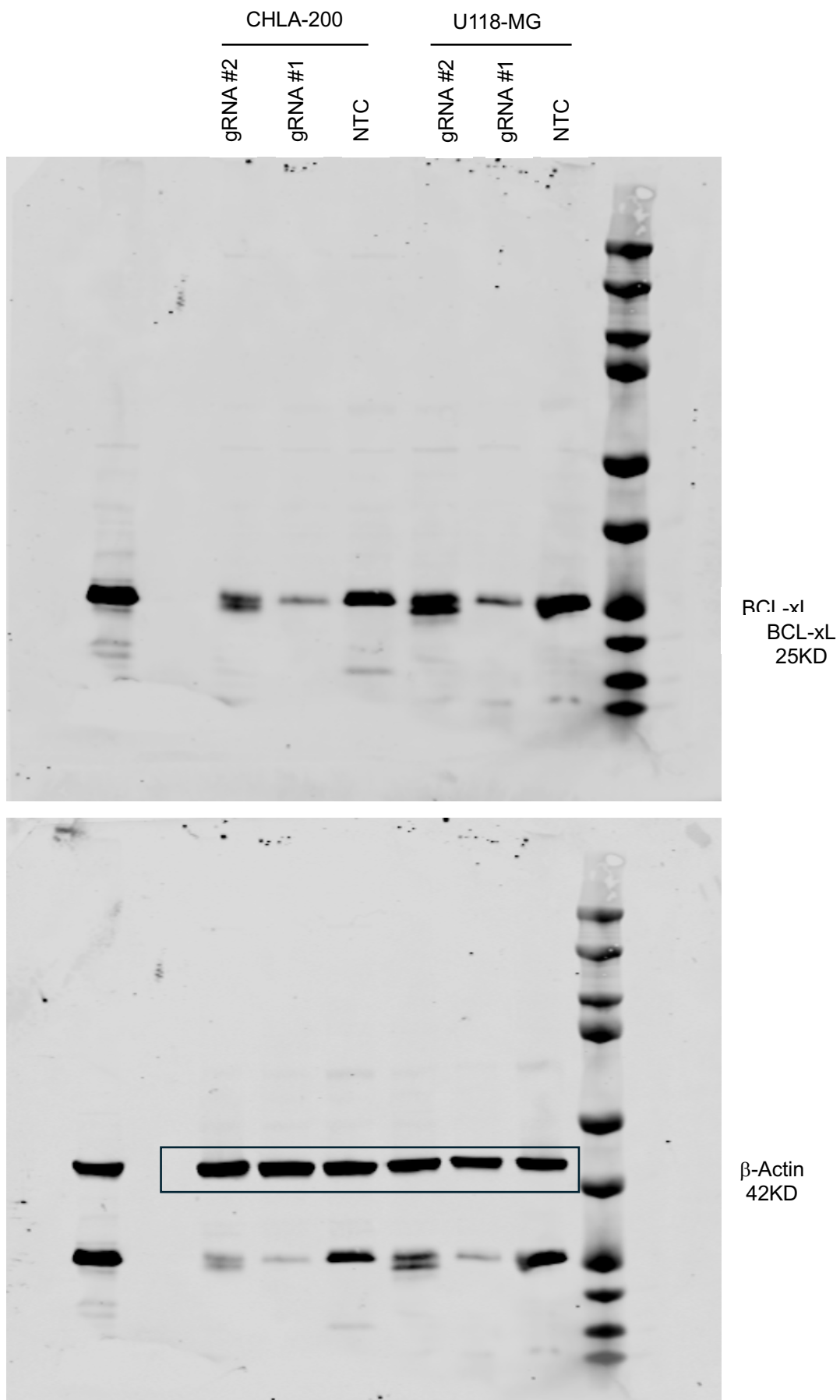

**Figure 4G**

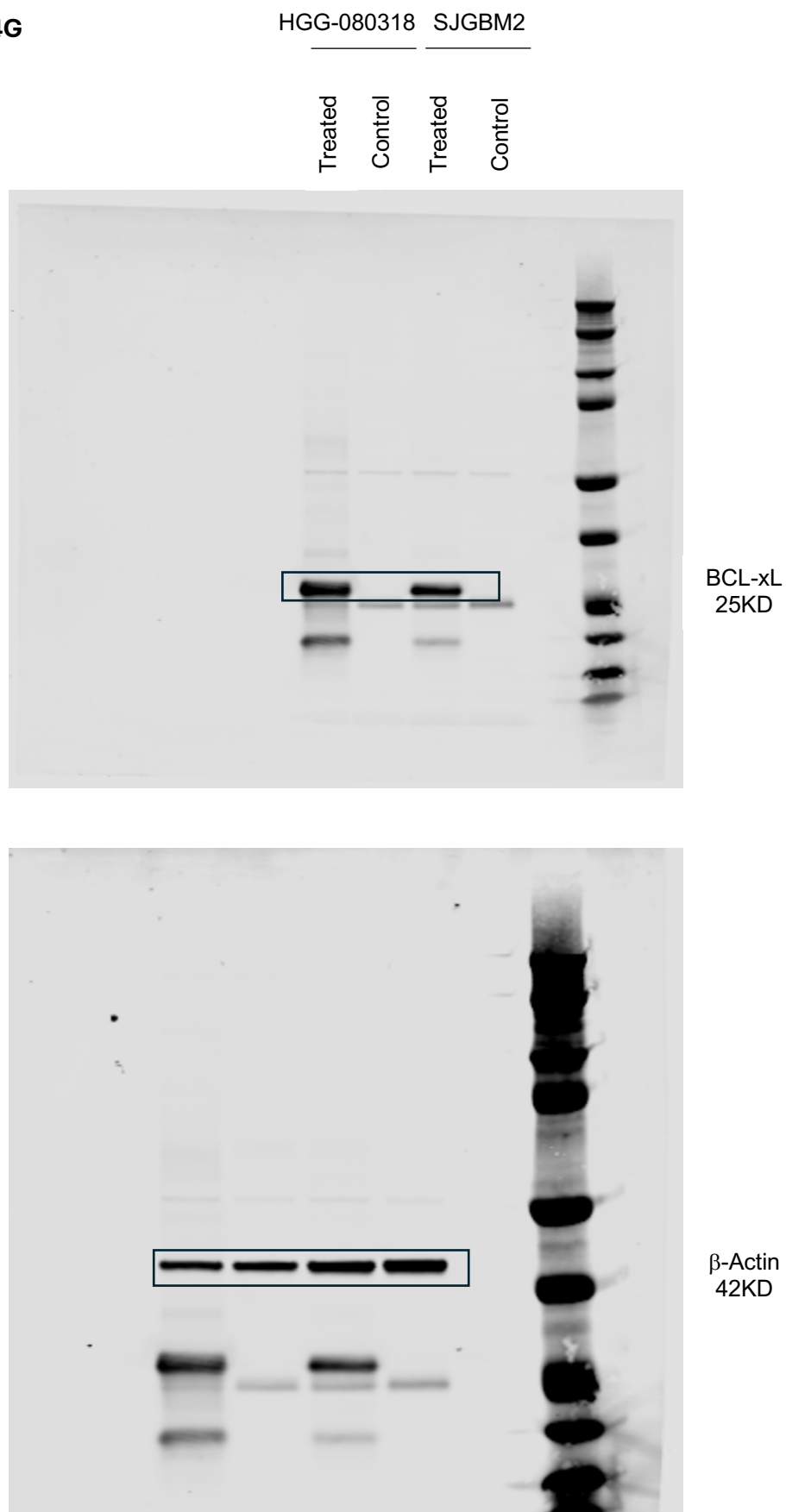

Supplement: Unedited blot and gel images [file jciinsight-10-184601-s046.pdf]
